# Simultaneous overactivation of Wnt/β-catenin and TGFβ signalling by miR-128-3p confers chemoresistance-associated metastasis in NSCLC

**DOI:** 10.1038/ncomms15870

**Published:** 2017-06-19

**Authors:** Junchao Cai, Lishan Fang, Yongbo Huang, Rong Li, Xiaonan Xu, Zhihuang Hu, Le Zhang, Yi Yang, Xun Zhu, Heng Zhang, Jueheng Wu, Yan Huang, Jun Li, Musheng Zeng, Erwei Song, Yukai He, Li Zhang, Mengfeng Li

**Affiliations:** 1 Department of Microbiology, Sun Yat-sen University Zhongshan School of Medicine, Guangzhou 510080, China; 2 Key Laboratory of Tropical Disease Control (Sun Yat-sen University), Ministry of Education, Guangzhou 510080, China; 3 Guangdong Engineering and Technology Research Center for Disease-Model Animals, Sun Yat-sen University, Guangzhou 510006, China; 4 Central Laboratory of The Eighth Affiliated Hospital of Sun Yat-sen University, Shenzhen 518033, China; 5 State Key Laboratory of Respiratory Diseases and Guangzhou Institute of Respiratory Diseases, The First Affiliated Hospital of Guangzhou Medical University, Guangzhou 510120, China; 6 State Key Laboratory of Oncology in South China, Department of Medical Oncology, Sun Yat-Sen University Cancer Center, Guangzhou 510060, China; 7 Department of Pharmacology, Zhongshan School of Medicine, Sun Yat-sen University, Guangzhou 510080, China; 8 Neurosurgery Intensive Care Unit, The First Affiliated Hospital of Sun Yat-sen University, Guangzhou 510080, China; 9 Department of Biochemistry, Sun Yat-sen University Zhongshan School of Medicine, Guangzhou 510080, China; 10 State Key Laboratory of Oncology in South China, Department of Experimental Research, Sun Yat-Sen University Cancer Center, Guangzhou 510060, China; 11 Department of Breast Surgery, Sun Yat-Sen Memorial Hospital, Sun Yat-Sen University, Guangzhou 510120, China; 12 Department of Medicine and Department of Biochemistry and Molecular Biology, Georgia Cancer Center, Augusta University, Augusta, Georgia 30912, USA

## Abstract

Cancer chemoresistance and metastasis are tightly associated features. However, whether they share common molecular mechanisms and thus can be targeted with one common strategy remain unclear in non-small cell lung cancer (NSCLC). Here, we report that high levels of microRNA-128-3p (miR-128-3p) is key to concomitant development of chemoresistance and metastasis in residual NSCLC cells having survived repeated chemotherapy and correlates with chemoresistance, aggressiveness and poor prognosis in NSCLC patients. Mechanistically, miR-128-3p induces mesenchymal and stemness-like properties through downregulating multiple inhibitors of Wnt/β-catenin and TGF-β pathways, leading to their overactivation. Importantly, antagonism of miR-128-3p potently reverses metastasis and chemoresistance of highly malignant NSCLC cells, which could be completely reversed by restoring Wnt/β-catenin and TGF-β activities. Notably, correlations among miR-128-3p levels, activated β-catenin and TGF-β signalling, and pro-epithelial-to-mesenchymal transition/pro-metastatic protein levels are validated in NSCLC patient specimens. These findings suggest that miR-128-3p might be a potential target against both metastasis and chemoresistance in NSCLC.

## Abstract (web summary)

Chemoresistance and metastasis are tightly associated features in non-small cell lung cancer (NSCLC). Here, the authors show that resistant NSCLCs have high levels of miR-128-3p which promotes EMT and cancer stem cell survival through the modulation of both Wnt/B-catenin and TGF-β pathway.

Distant metastasis is responsible for more than 90% of cancer-related deaths^1^. In non-small cell lung cancer (NSCLC), it is estimated that >50% of patients show evidence of distant metastasis at the time of diagnosis, and only ∼1% of patients with metastatic NSCLC survive 5 or more years after the diagnosis of metastases, with a median survival time of 7 months^2^. The current first-line treatment for the majority of metastatic NSCLC in the clinic remains limited to platinum-based chemotherapy, which is frequently accompanied by the rapid development of drug resistance. Although other chemotherapeutic drugs are suggested as a second-line treatment, pan-chemoresistance to all chemotherapeutic agents occurs almost invariably, ultimately causing therapeutic failure and uncontrolled disease progression^3^.

Tumour metastasis and chemoresistance are frequently revealed in late-stage cancers as two major inseparable causes of lethality. Biologically, tumour metastasis occurs when tumour cells are modified by cellular programs, including the epithelial-to-mesenchymal transition (EMT), which is characterized by the loss of epithelial differentiation and the acquisition of the mesenchymal phenotype^1^. On the other hand, the emergence of chemoresistance results when tumour cells initiate auto-protective programming to survive the pressure of cell death-inducing chemotherapeutic agents. Despite having been studied separately in the past, accumulating evidence suggests that tumour metastasis and chemoresistance not only commonly present simultaneously clinically but might also be intrinsically associated biological events^4^,^5^. It was observed, for example, that NSCLC patients with stage IV disease exhibit a substantially lower overall response rate to chemotherapy than patients with locally advanced disease^6^,^7^, suggesting that metastatic NSCLC patients are prone to be more resistant to chemotherapy in the clinic. In parallel, several biological events causing concurrent tumour metastasis and chemoresistance have been reported^8^,^9^. Recently, a mechanism characterized by an interaction between the host microenvironment and cancer cells, thereby linking chemotherapy failure with metastatic relapse, was characterized in a study on breast cancer^10^. Despite these observations, the molecular as well as cellular mechanisms underlying the connection between metastasis and chemoresistance, which may vary among different cancer types and clinical contexts, have yet to be uncovered.

The recent recognition of a potentially significant contribution of stemness-possessing malignant cells in cancer lesions, or cancer stem cells (CSCs), to tumour relapse and cancer cell dissemination, as well as to the development of resistance to chemotherapy or radiation therapy, has provided important clues to better understand the malignant properties of human cancers^11^. For example, Mani *et al*.^12^ reported that a CSC-like state of differentiated cancer cells can be induced by EMT programming, suggesting that CSCs may underlie the systemic dissemination from the primary tumour mass by acquiring mesenchymal features. In parallel, an increasing number of studies have demonstrated that EMT plays a critical role not only in tumour metastasis but also in drug resistance and tumour recurrence, and it is tightly linked with the biological properties of CSCs^13^,^14^. Many lines of evidence have indicated that the formation of the CSC population and EMT programming in tumours can be regulated by genetic alterations or epigenetic regulation, and the relative contribution of genetic versus epigenetic mechanisms to the metastatic and drug-resistant phenotype most likely varies in different cancer types and biological scenarios. Ultimately, the mechanistic foundation for the development of CSC and EMT phenotypic properties in NSCLC cells remains poorly understood.

The combined importance of chemoresistance and metastasis prompted us to investigate the molecular basis by which chemoresistant cancer cells maintain their stem cell characteristics and acquire metastatic traits. Here, we establish a chemoresistance-associated metastasis model of NSCLC xenografts and demonstrate that miR-128-directed CSC and EMT programming in various NSCLC cell lines confers tumourigenesis, metastasis and chemoresistance by simultaneously activating the β-catenin and TGF-β signalling. Importantly, the effect of miR-128-3p antagonism on impairing both chemoresistance and metastasis in highly malignant NSCLC cells could be reversed by re-activation of the Wnt/β-catenin and TGF-β signalling. Our findings therefore implicate a possibility of simultaneously suppressing the two pathways by targeting one molecule to overcome chemoresistance and metastasis in NSCLC.

## Results

### miR-128-3p is a chemoresistance-assoicated miRNA in NSCLC

**Figure 1:**
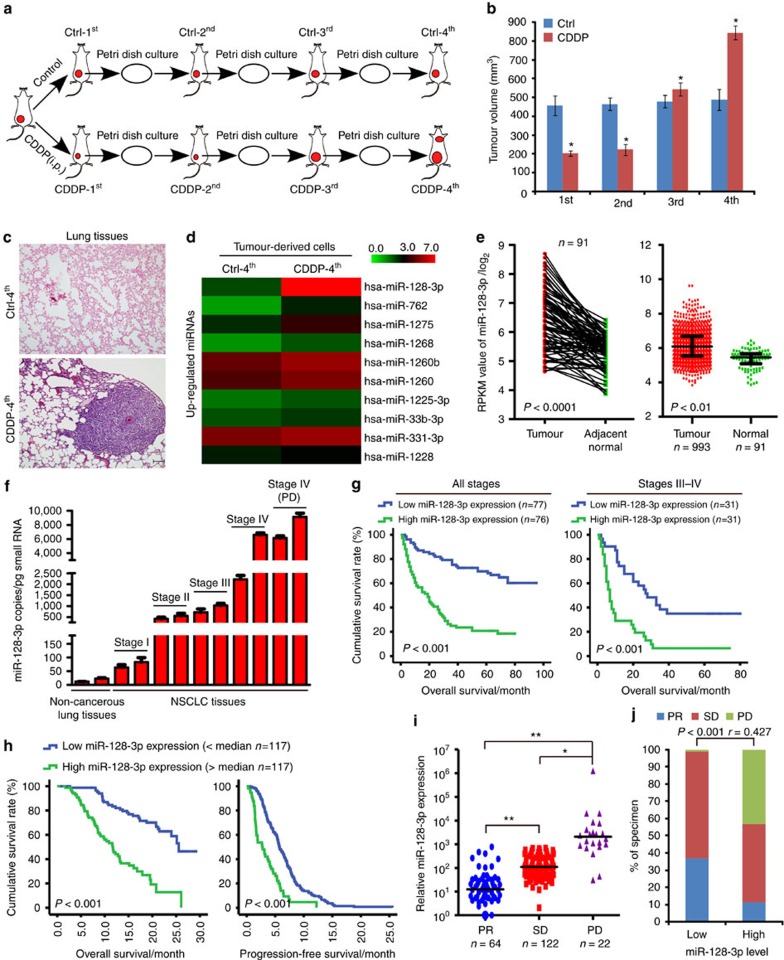
miR-128-3p overexpression in NSCLC specimens correlates with poor patient survival, disease progression and chemotherapy failure. (**a**) Schematic representation for the establishment of a chemoresistant subcutaneous tumour model. Mice bearing A549-luc xenografts were intraperitoneally injected with the first round of CDDP treatment (CDDP-1st) or control treatment (Ctrl-1st), and cells were isolated from the resultantly remaining tumours, cultured and re-transplanted, followed by a second round of CDDP treatment (CDDP-2nd) or control treatment (Ctrl-2nd) and so ons. (**b**) Tumour volume of subcutaneous tumours at the indicated rounds of CDDP or Ctrl treatment. (**c**) Lung tissues obtained from mice bearing NSCLC xenografts at the fourth round of Ctrl and CDDP treatment were histologically examined. Scale bar, 100 μm. (**d**) Tumour-derived cultured cells from Ctrl-4th and CDDP-4th treatment were subjected to miRNA array analysis, revealing the top 10 upregulated miRNAs in chemoresistant, metastatic A549-luc-CDDP-4th cells. Intensity values representing miRNA expression levels were log10 transformed. (**e**) Expression of miR-128-3p in human lung cancer clinical specimens using the TCGA microRNA Hiseq expression array data. (**f**) Absolute real-time PCR using a standard curve of miR-128-3p expression in adjacent normal lung tissues and the indicated NSCLC specimens. Error bars represent mean±s.d. derived from three independent experiments. A two-tailed Student’s *t*-test was used for statistical analysis (*P<0.05). (**g**) Kaplan–Meier analysis of OS of a cohort of 153 NSCLC patients at all stages and late stages III–IV. Each subgroup was divided into the low- (below or equal to the median value) and high-miR-128-3p expression groups (above the median value). (**h**) Kaplan–Meier analysis of OS and PFS of a cohort of 234 NSCLC patients (stage I, *n*=27; stage II, *n*=65; stage III, *n*=52; stage IV, *n*=90) receiving CDDP-based first-line treatment who were divided into high (>median, *n*=117) and low (<median, *n*=117) miR-128-3p expression groups. (**i**) Expression levels of miR-128-3p in NSCLC patients subgrouped by curative responsiveness to chemotherapy, including PR, SD and PD, according to the RECIST. Ordinate utilizes the logarithmic scale (log10). *P<0.05, **P<0.01. (**j**) Correlation of miR-128-3p expression level with curative responsiveness of NSCLC patients to chemotherapy. RECIST, Response Evaluation Criteria in Solid Tumours.

In our chemoresistant tumour model, A549-luc cell xenografts developed chemoresistance in the third round of cis-diaminedichloroplatinum (CDDP) treatment (CDDP-3rd) and chemoresistance-associated metastases following the fourth round of treatment (Fig. 1a–c and Supplementary Fig. 1a–c). Upon examining differential miRNA profiles between tumour-derived cultured cells from the Ctrl-4th and CDDP-4th treatments, miR-128-3p was among the most markedly upregulated miRNAs in chemoresistant, metastatic A549-luc-CDDP-4th cells (Fig. 1d and Supplementary Fig. 1d) and found to be highly expressed in a panel of NSCLC cell lines and a naturally highly metastatic murine lung cancer cell line LL/2-luc-M38 (Supplementary Fig. 1e). miR-128-3p was further verified to be highly expressed in residual tumours resistant to CDDP-3rd and CDDP-4th treatments (Supplementary Fig.1f). As several independent databases show that miR-128-3p is the predominantly expressed miRNA species, we sought to investigate the potential role of miR-128-3p in the progression of NSCLC. Notably, only the miR-128-3p species is significantly upregulated in NSCLC as compared with that in normal tissue specimens in the The Cancer Genome Atlas (TCGA) public lung cancer data sets (Fig. 1e). This result is consistent with a published large-scale, detailed analysis of the miRNA profiles in 540 specimens of six types of solid tumours, which showed that miR-128-3p is among the most commonly upregulated microRNAs in lung cancer and also highly expressed in colon and pancreatic cancers^15^. Interestingly, low-dose CDDP treatment, which generally does not cause cell death, failed to induce, whereas CDDP treatment at the IC_50_ concentration induced miR-128-3p expression in NSCLC cells (Supplementary Fig. 1g). Moreover, miR-128-3p expression was significantly upregulated in the surviving NSCLC cells after continuous treatment of CDDP at a high-dose (7 μg ml^−1^) in A549 and H520 cells for 7 days (Supplementary Fig. 1h), suggesting that pre-existing subsets of treatment-naive NSCLC cells with high miR-128-3p levels might be prone to selection during chemotherapeutic treatment and miR-128-3p upregulation might be associated with the observed chemoresistance and the linked metastasis of NSCLC xenografts. In consistence, absolute quantification by real-time RT-PCR showed that the absolute miR-128-3p levels were significantly higher in late-stage NSCLC (716–6,572 copies per pg small RNA) or metastatic tissue resistant to clinical chemotherapy (6,133–9,146 copies) than those in normal lung (12–23 copies) or early-stages NSCLC tissue (64–550 copies; Fig. 1f).

Importantly, miR-128-3p expression in a cohort of 153 NSCLC patients, which were divided into a high (>median, *n*=76) and a low miR-128-3p expression group (≤median, *n*=77), significantly correlated with NSCLC clinical staging and Tumour–Node–Metastasis classification (Supplementary Fig. 2a). Kaplan–Meier analysis demonstrated that the median overall survival (OS) time of patients with high miR-128-3p expression was only 19.0 months, whereas that for those with low miR-128-3p expression was 68.0 months (log rank test, *P*<0.001) (Fig. 1g). Such significant differences were further observed in NSCLC patients of early stage II and late stages III–IV subgroups (Fig. 1g and Supplementary Fig. 2b); a high level of miR-128-3p expression was found to be associated with a poor prognosis in all histological subtypes stratified in the enroled NSCLC patients (Supplementary Fig. 2c). Moreover, Cox’s multivariate survival analysis showed that miR-128-3p level was an independent prognostic factor for NSCLC patients (Supplementary Fig. 2d). Furthermore, the association between high miR-128-3p expression with shorter OS and progression-free survival (PFS) was validated in another independent cohort of 234 cases of NSCLC patients receiving multiple rounds of CDDP-based first-line therapy. Patients with high-level miR-128-3p expression (*n*=117) had a median OS time of 11.7 months, and revealed disease progression with a median PFS time of 2.7 months, in contrast to the 25.5-month median OS time and the 5.7-month median PFS time for patients with low miR-128-3p expression (Fig. 1h). Interestingly, miR-128-3p level was lower in patients showing partial response (PR) than in those presenting progressive disease (PD) or stable disease (SD) (Fig. 1i,j; the full description for PR, PD and SD is provided in the Methods), suggesting that high miR-128-3p expression clinically correlated with poor curative responsiveness to CDDP-based therapy for NSCLC patients.

### miR-128-3p induces EMT and CSC programming in NSCLC cells

**Figure 2:**
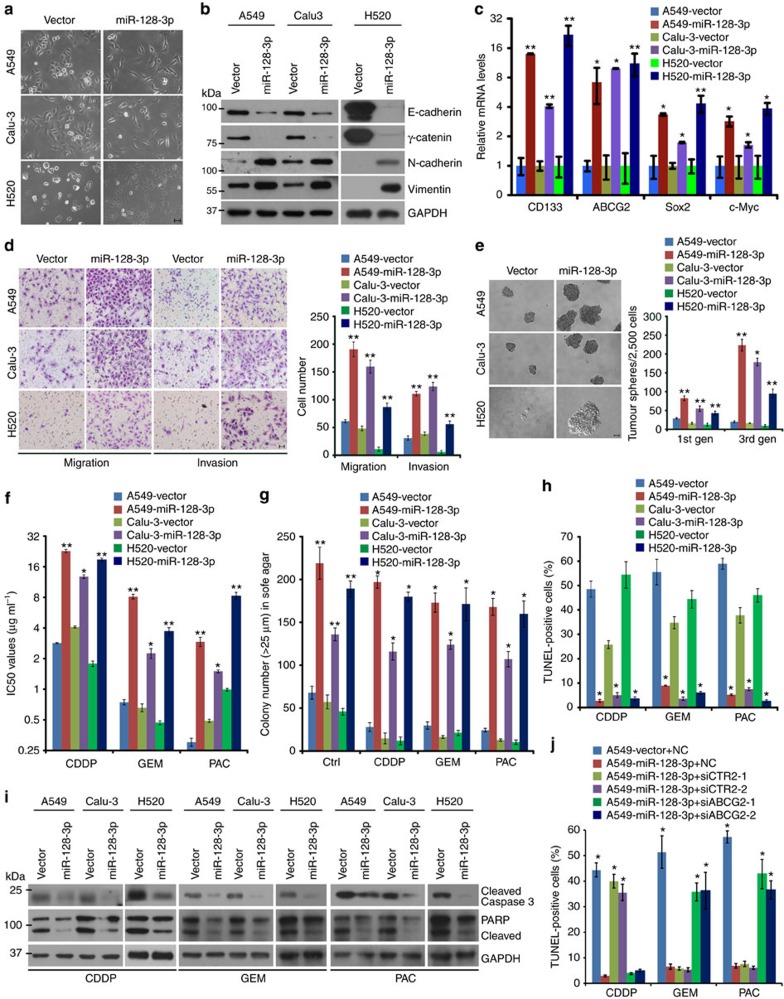
miR-128-3p induces a phenotype of EMT and CSC in NSCLC cells. (**a**) Phase-contrast images show the morphology of indicated NSCLC cells overexpressing miR-128-3p or control vector. Scale bar, 50 μm. (**b**) WB analysis was used to examine expression levels of pro-EMT markers (decreased epithelial markers such as E-cadherin and γ-catenin and increased mesenchymal markers such as N-cadherin and Vimentin) in the indicated cells. (**c**) qRT-PCR was used to measure relative expression of stemness-related genes, including Sox2, Myc, CD133 and ABCG2, in the indicated cells. (**d**) Representative images and average cell number of invading or migratory cells. Scale bar, 50 μm. (**e**) Representative images and quantification of non-adherent tumour spheres during three consecutive passages seeded by the indicated cells. Scale bar: 100 μm. (**f**) Cell viability assay of the indicated cells treated with CDDP, gemcitabine (GEM) and paclitaxel (PAC), respectively, at various concentrations to measure their respective IC50 values. (**g**) Quantification of colonies counted in anchorage-independent growth ability assay in the presence of CDDP (3 μg ml^−1^), gemcitabine (GEM, 0.5 μg ml^−1^), paclitaxel (PAC, 0.5 μg ml^−1^) or control solvent (Ctrl) treatment. (**h**) Quantification of TUNEL staining in indicated cells after CDDP (3 μg ml^−1^) gemcitabine (GEM, 0.5 μg ml^−1^), and paclitaxel (PAC, 0.5 μg ml^−1^) treatment, respectively. The numbers of TUNEL-positive cells were counted from 10 random fields and presented as percentages of total cell numbers. (**i**) WB analysis for proteolytic cleavage of Caspase-3 and PARP in indicated cells after indicated treatment. (**j**) Quantification of the percentages of TUNEL-positive cells in indicated cells transfected with negative control siRNAs (NC), CTR2 siRNAs or ABCG2 siRNAs after CDDP, gemcitabine (GEM), and paclitaxel (PAC) treatment, respectively. Error bars represent mean±s.d. derived from three independent experiments. A two-tailed Student’s *t*-test was used for statistical analysis (*P<0.05, **P<0.01).

To address whether miR-128-3p might play a key role in NSCLC malignancy, a pri-miR-128-2-expressing construct containing sequences of both miR-128-3p and miR-128-2-5p were transduced into NSCLC cells, where only miR-128-3p levels were markedly upregulated, whereas miR-128-2-5p levels were slightly increased as compared to vector-control NSCLC cells and were far lower than miR-128-3p levels (Supplementary Fig. 3a). Of note, overexpressing ectopic miR-128-3p to the absolute level comparable to that in human NSCLC tissue (Fig. 1f and Supplementary Fig. 3a) in two NSCLC cell lines, A549 and Calu-3, which expresses moderate levels of miR-128-3p, and in a lung squamous cell carcinoma line, NCI-H520 (H520), which expresses a relatively low level of miR-128-3p (Supplementary Fig. 1e), induced EMT and CSC-like traits, including scattered spindle-shaped morphology (Fig. 2a), increased pro-EMT markers (Fig. 2b and Supplementary Fig. 3b), increased transcription of biomarkers related to cancer stemness (Fig. 2c) and an increased proportion of CD133^+^ NSCLC cells (Supplementary Fig. 3c). Furthermore, miR-128-3p was upregulated in tumour sphere-derived NSCLC cells, compared with their parental cells (Supplementary Fig. 3d). Consistent with these alterations, miR-128-3p-transduced NSCLC cells acquired significantly higher migratory and invasive abilities than the vector-control cells (Fig. 2d and Supplementary Fig. 3e). In parallel, miR-128-3p overexpression greatly potentiated the self-renewal ability of the NSCLC cells to grow into more, larger-sized non-adherent cell spheres and increased the proportions of stem cell-like SP fractions (Fig. 2e and Supplementary Fig. 3f). Meanwhile, various NSCLC cell lines overexpressing miR-128-3p exhibited increased IC_50_ values for cisplatin, gemcitabine and paclitaxel and survived to form far more and larger cellular colonies when treated with these chemotherapeutic drugs, as compared with their respective vector-control cells (Fig. 2f,g). The increased survival of miR-128-3p-overexpressing NSCLC cells under drug treatment was closely associated with miR-128-3p-induced anti-apoptotic effect, as demonstrated by miR-128-3p-induced decrease of TUNEL-positive cells and suppression of Caspase 3 and PARP activation (Fig. 2h,i), as well as abrogation of cellular uptake of the chemotherapeutic drugs (Supplementary Fig. 3h). Furthermore, we found that in addition to inducing expression of ABCG2, a cancer stemness marker and multi-drug transporter as aforementioned, miR-128 also upregulated expression of another drug transporter CTR2 (Supplementary Fig. 3g), silencing of which has been extensively reported to promote uptake of platinum-based drugs and cisplatin-induced cellular apoptosis^16^,^17^. Of note, silencing CTR2 in miR-128-3p-overexpressing NSCLC cells reversely increased cisplatin uptake and cisplatin-induced apoptosis, whereas it hardly altered uptake of gemcitabine or paclitaxel, or cellular apoptosis induced by these two chemotherapeutic drugs (Fig. 2j and Supplementary Fig. 3g–i). Interestingly, silencing ABCG2 in miR-128-3p-overexpressing NSCLC cells enhanced the intake of gemcitabine and paclitaxel and cellular apoptosis induced by the two drugs but did not influence cisplatin uptake or cisplatin-induced apoptosis (Fig. 2j and Supplementary Fig. 3g–i), suggesting that various drug transporter proteins might together contribute to miR-128-3p-induced chemoresistance in NSCLC cells. Notably, we also observed that overexpression of miR-128-3p significantly accelerated NSCLC cell proliferation and survival in the three-dimensional and two-dimensional settings (Fig. 2g and Supplementary Fig. 3j). However, although miR-128-3p upregulated the expression of cyclin D1 (CCND1), a critical driver for tumour cell proliferation, silencing cyclin D1 in miR-128-3p-overexpressing NSCLC cells barely influenced their ability to migrate, whereas significantly deteriorated their cell proliferation and survival (Supplementary Fig. 3j–l), suggesting that the observed miR-128-3p-induced migration is not affected by its promotion of cellular proliferation and survival.

In contrast, NSCLC cells silenced for endogenous miR-128-3p using anti-miR-128-3p oligonucleotides (Anti-miR) expressed decreased levels of pro-EMT and stemness-determinant genes (Supplementary Fig. 4a–c), and revealed remarkably compromised abilities to migrate, invade and undergo progressive wound healing (Supplementary Fig. 4d,e), along with weakened self-renewal ability and reduced SP cell proportions as compared with negative control (NC) cells (Supplementary Fig. 4f,g). Moreover, inhibition of miR-128-3p decreased the IC_50_ values for cisplatin, gemcitabine and paclitaxel, increased cellular drug uptake, reduced expression of drug transporters CTR2 and ABCG2, and promoted cellular apoptosis induced by these chemotherapeutic drugs in NSCLC cells (Supplementary Fig. 4h–l). Taken together, these data demonstrate a remarkable role of miR-128-3p in the maintenance of EMT and CSC programming and induction of drug resistance in NSCLC cells.

### miR-128-3p confers tumorigenicity and metastasis *in vivo*

**Figure 3:**
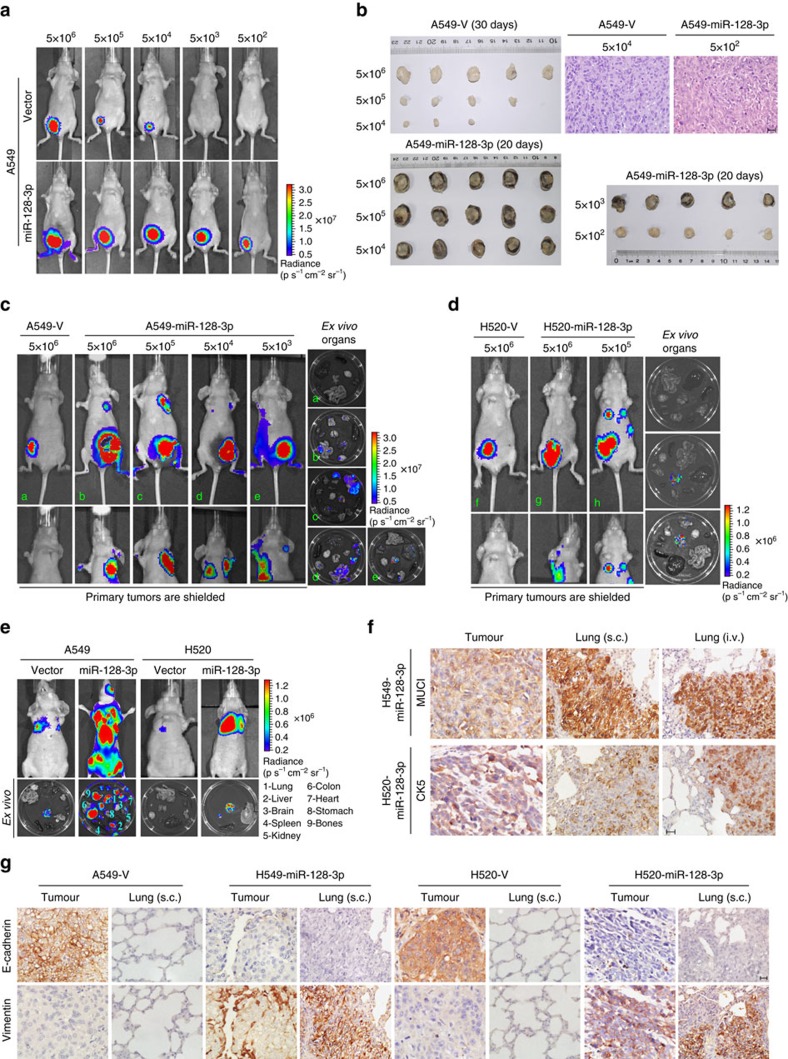
miR-128-3p overexpression promotes tumorigenesis and spontaneous/systemic metastasis of NSCLC xenografts *in vivo*. (**a**) A549-luc-Vector and A549-luc-miR-128-3p cells of the indicated dosages were, respectively, implanted in the inguinal folds of different nude mice. Representative bioluminescent images of subcutaneous tumour outgrowth are shown. (**b**) Subcutaneous tumours formed by the indicated cells were dissected and imaged. H&E histologically confirmed tumour cells. Scale bar, 25 μm. (**c** and **d**) For the spontaneous metastasis model, bioluminescent images of subcutaneous tumours of the indicated cells, distant metastasis signals (with tumours shielded), and *ex vivo* organ metastases are shown. (**e**) For the experimental metastasis model, bioluminescent images of systemic metastases and *ex vivo* organ metastases including those in the lungs, liver, spleen, kidney, colon, heart, stomach, bones and brain, are shown. (**f**) Immunostaining for the lung adenocarcinoma marker mucin 1 (MUC1) and lung squamous cell carcinoma marker cytokeratin 5 (CK5), respectively, in spontaneous and experimental lung metastatic lesions developed by subcutaneous inoculation (s.c.) and intravenous injection (i.v.) of the indicated cells. Scale bar, 25 μm. (**g**) Immunostaining of two key EMT biomarkers, E-cadherin and Vimentin, in primary subcutaneous tumour tissues and lung metastatic lesions. Scale bar, 25 μm. H&E, haematoxylin and eosin.

In the tumorigenicity experiment, as few as 5 × 10^2^ and 5 × 10^3^ A549-luc-miR-128-3p and H520-luc-miR-128-3p cells, respectively, were able to form detectable tumours, whereas corresponding vector-control cells generated visible tumours only when inoculated with more than 5 × 10^4^ cells and formed markedly smaller tumours than miR-128-3p-overexpressing cells (Fig. 3a,b and Supplementary Fig. 5a–d). Moreover, mice bearing A549-luc-miR-128-3p or H520-luc-miR-128-3p tumours derived from various cell concentrations exhibited spontaneous metastatic colonization in multiple distal organs, in contrast to the vector-control group showing no distant metastases (Fig. 3c,d and Supplementary Fig. 5b–d). Of note, by continuously extending the experimental time for 1 more month for tumour growth in the A549-Vector xenografts group, which reached as large as that of A549-miR-128 cell xenografts, there was still no visible metastasis derived from the resultant tumours (Supplementary Fig. 5e). In parallel, silencing Cyclin D1 in miR-128-overexpressing NSCLC cells barely influenced their ability to metastasize, whereas significantly deteriorated their primary tumour growth (Supplementary Fig. 5e), further suggesting that miR-128-induced metastasis may be unreadily affected by its promoting effect on cell proliferation. In the experimental metastasis model, intravenously injecting A549-luc-miR-128-3p or H520-luc-miR-128-3p cells caused prominent metastases in various distal organs, whereas A549-luc-Vector and H520-luc-Vector cells caused only limited pulmonary metastases (Fig. 3e and Supplementary Fig. 6a–d). Importantly, tumour xenografts, spontaneous and experimental lung metastatic lesions developed by subcutaneous inoculation (s.c.) or intravenous injection (i.v.) of A549-miR-128-3p cells, respectively, as well as their metastatic lesions in multiple organs, were positive for MUC1 staining, while tumour xenografts and lung metastatic lesions of H520-miR-128-3p cells were also positive for CK5 staining, suggesting that these metastatic lesions are of epithelial origin from the corresponding inoculated NSCLC cells (Fig. 3f and Supplementary Fig. 6b). Furthermore, xenografts, as well as their lung metastatic lesions derived from A549-miR-128-3p and H520-miR-128-3p cells exhibited lower E-cadherin and higher Vimentin levels than respective vector-control xenografts (Fig. 3g), strengthening the notion that EMT is an important event key to the miR-128-3p-induced lung metastases. Collectively, these data suggest that miR-128 is capable of conferring tumorigenicity and spontaneous, systemic metastasis of NSCLC cells *in vivo*.

### Therapeutic effect of miR-128-3p antagonism *in vivo*

**Figure 4:**
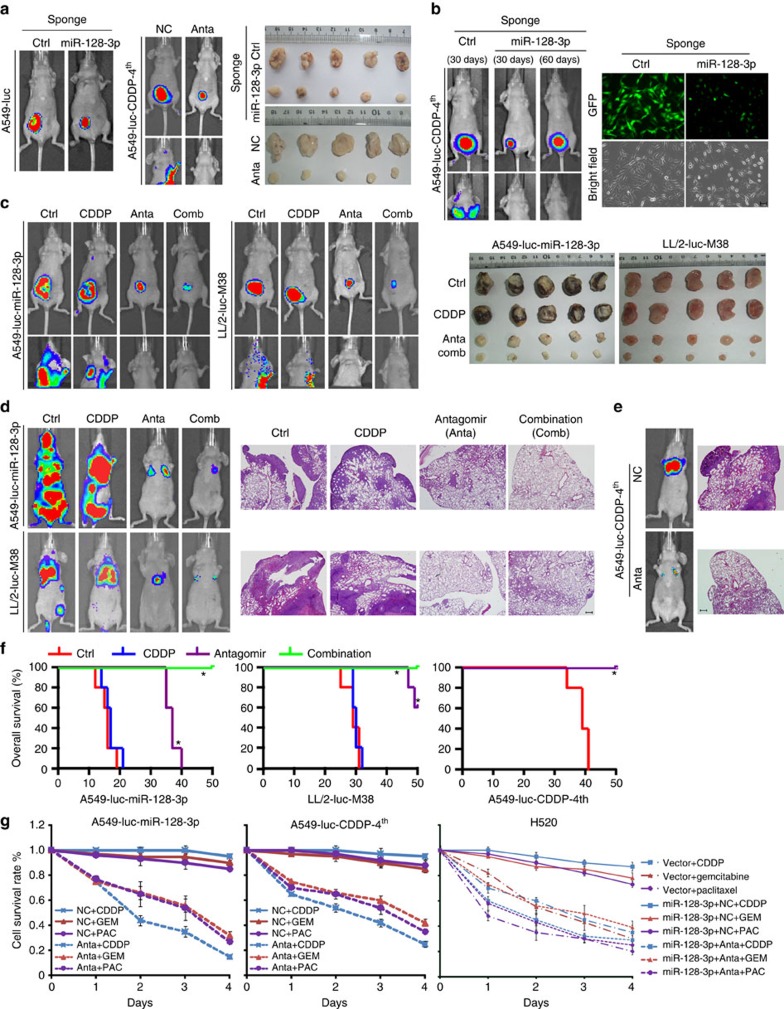
Inhibiting miR-128-3p suppresses tumour growth and distant metastasis and reverses resistance of highly malignant NSCLC cells to chemotherapeutic drugs. (**a**) A549-luc-control-sponge and A549-luc-miR-128-3p-sponge cells were, respectively, implanted in the inguinal folds of separate nude mice. On day 45, tumours were dissected and imaged as shown. A549-luc-CDDP-4th cells were subcutaneously implanted, followed by intravenous administration of control (NC) or miR-128-3p antagomirs (Anta), respectively. On day 30, tumours were dissected and imaged as shown. Bioluminescent images of subcutaneous tumours or/and spontaneous metastasis are shown. (**b**) A group of mice subcutaneously inoculated with vector-control A549-luc-CDDP4th cells were killed at day 30 after the initial inoculation, and a parallel group of mice were inoculated with A549-luc-CDDP4th cells stably silenced with miR-128-3p by the miR-128-3p sponge and killed for further analysis at day 60 after the initial inoculation. Bioluminescent images of subcutaneous tumours or/and spontaneous metastases are shown (left). Bright-field imaging and fluorescent visualization confirmed efficient inhibition of miR-128-3p by miRNA sponge strategy (right panel). Scale bar, 50 μm. (**c**) Bioluminescent images of subcutaneous tumours and spontaneous metastasis of A549-luc-miR-128-3p or LL/2-luc-M38 cells in response to CDDP treatment or its combination (Comb) with miR-128-3p antagomir (Anta). Corresponding residual tumours were dissected and imaged as shown. (**d** and **e**) In response to the indicated treatments, bioluminescent images of experimental distant metastasis of the indicated cells are shown. Lung metastases were histologically confirmed by H&E staining. Scale bar, 200 μm. (**f**) OS of mice in the experimental metastasis model receiving the indicated treatments. *P<0.05. (**g**) Cell viability assays determined the resistance or sensitivity to chemotherapeutic drugs including CDDP, gemcitabine (GEM) and paclitaxel (PAC), which administered, respectively, at a final concentration of 3, 0.5 and 1 μg ml^−1^, were separately added to the indicated cells pretreated with 100 nM control (NC) or miR-128-3p antagomir (Anta). H&E, haematoxylin and eosin.

To evaluate the pathophysiological function of endogenous miR-128-3p, miRNA sponge and antagomir were used as antagonists to suppress miR-128-3p. Transduction of the miR-128-3p sponge competitively reduced the availability of miR-128-3p, as demonstrated by stably decreased GFP reporter signal, and drastically inhibited the growth of A549 tumours (Fig. 4a and Supplementary Fig. 7a). In parallel, intravenous administration of miR-128-3p antagomir strikingly diminished primary tumour growth and spontaneous/systemic metastasis of the subcutaneously inoculated or intravenously injected chemoresistant metastatic cells A549-luc-CDDP-4th (Fig. 4a,e and Supplementary Fig. 7b,f). Importantly, the control group bearing xenografts of vector-control A549-luc-CDDP4th cells showed remarkable distant metastases in 30 days; in contrast, the other group bore reduced primary tumours of miR-128-3p-silenced A549-luc-CDDP4th cells in 30 days and presented no sign of distant metastases even in the additional 30 days of observation although the resultant primary tumours reached a size comparable to that of the control group near experiment endpoint (Fig. 4b and Supplementary Fig. 7c), suggesting that the strong suppressive effect of antagonizing miR-128-3p on metastasis is basically due to its substantial reduction in metastatic capacity of NSCLC cells.

Furthermore, subcutaneous inoculation or intravenous injection of A549-luc-miR-128-3p or LL/2-luc-M38 cells, a highly malignant murine lung cancer cell line expressing high-level miR-128-3p (Supplementary Fig. 1e), revealed a complete resistance to CDDP treatment but a strong response to miR-128-3p antagomir, that is, displaying remarkable inhibition of primary tumour growth as well as spontaneous/systemic metastasis, and importantly such a suppressive effect on tumorigenesis and metastasis could be further enhanced when the miR-128-3p antagomir was used in combination with CDDP (Fig. 4c,d and Supplementary Fig. 7d,e). Moreover, miR-128-3p antagomir, or combined CDDP treatment, significantly improved the survival of mice bearing experimental metastases of A549-luc-miR-128-3p, LL/2-luc-M38 or A549-luc-CDDP-4th cells, compared with the corresponding controls or single CDDP treatment (Fig. 4f). In addition, A549-luc-miR-128-3p, A549-luc-CDDP-4th and H520-luc-miR-128-3p cells were resistant to CDDP, gemcitabine and paclitaxel treatment but can be re-sensitized by pretreatment with miR-128-3p antagomir (Fig. 4g), indicating that miR-128-3p might represent a valid target for reversing chemoresistance and associated distant metastasis.

### miR-128-3p overactivates the β-catenin and TGF-β pathways

**Figure 5:**
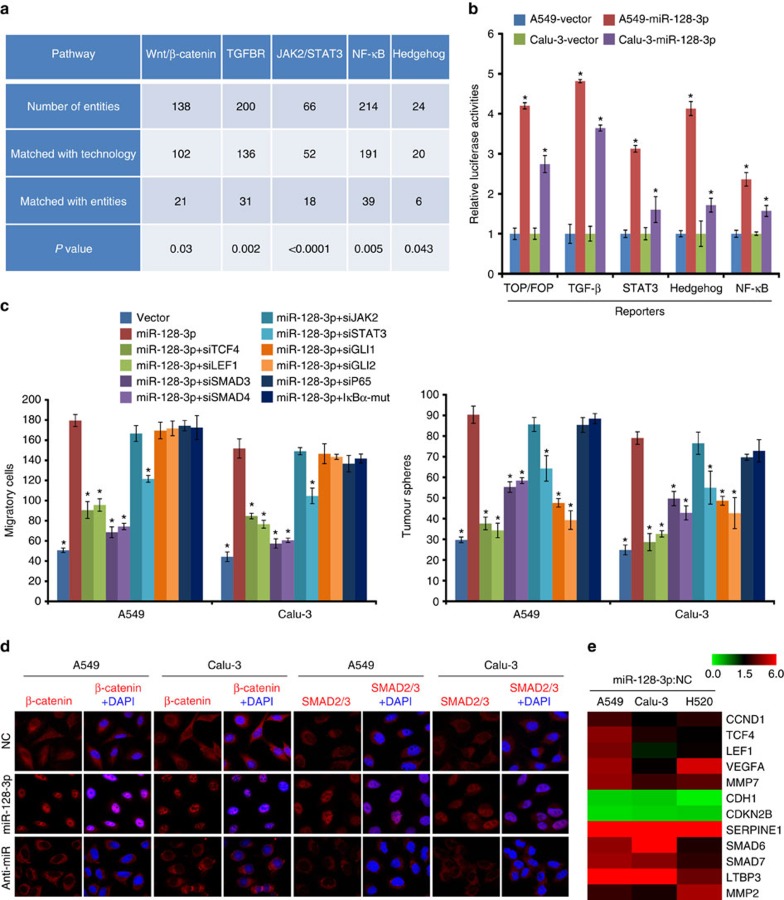
miR-128-3p activates Wnt/β-catenin and TGF-β signalling. (**a**) Effect of miR-128-3p overexpression on activation of significant pathways by microarray analysis. (**b**) Relative luciferase activities of the TOPflash/FOPflash, TGF-β, STAT3, Hedgehog and NF-κB signalling reporters in the indicated cells. (**c**) Effects of silencing the TOP/FOP, TGF-β, STAT3, Hedgehog and NF-κB signalling using the indicated treatments on the migration and tumour sphere growth of miR-128-3p-overexpressing A549 and Calu-3 cells. (**d**) Immunofluorescent staining shows subcellular SMAD2/3 or β-catenin localization in the indicated cells. Original magnification, × 630. (**e**) Heat map shows the qRT-PCR results of the upregulated downstream target genes of either β-catenin or TGF-β signalling in the indicated cells. Pseudo-colour scale values were log2 transformed. Error bars represent mean±s.d. derived from three independent experiments. A two-tailed Student’s *t*-test was used for statistical analysis (*P<0.05, **P<0.01). qRT-PCR, quantitative PCR with reverse transcipode.

Comparative mRNA profiling of A549-miR-128-3p and vector-control cells showed that several tumour progression-related pathways, including the Wnt/β-catenin, TGF-β, JAK2/STAT3, Hedgehog and NF-κB pathways, were likely to be activated by miR-128-3p overexpression, which were further validated in A549 and Calu-3 cells (Fig. 5a,b). When these five pathways were separately inhibited in experimental settings, only inhibition of β-catenin or TGF-β signalling could consistently impair the effects of miR-128-3p on the migration and self-renewal of NSCLC cells (Fig. 5c), suggesting that the biological effects of miR-128-3p were attributable to altered β-catenin and TGF-β signalling. In consistence, β-catenin and SMAD2/3 were found to be predominantly accumulated in the nucleus of miR-128-3p-transduced cells and localized in the cytoplasmic compartments of miR-128-3p-silenced cells (Fig. 5d). Consequently, miR-128-3p overexpression promoted, but miR-128-3p silencing inhibited, the transcription of several well-recognized downstream genes of the β-catenin and the TGF-β signalling (Fig. 5e and Supplementary Fig. 8a).

**Figure 6:**
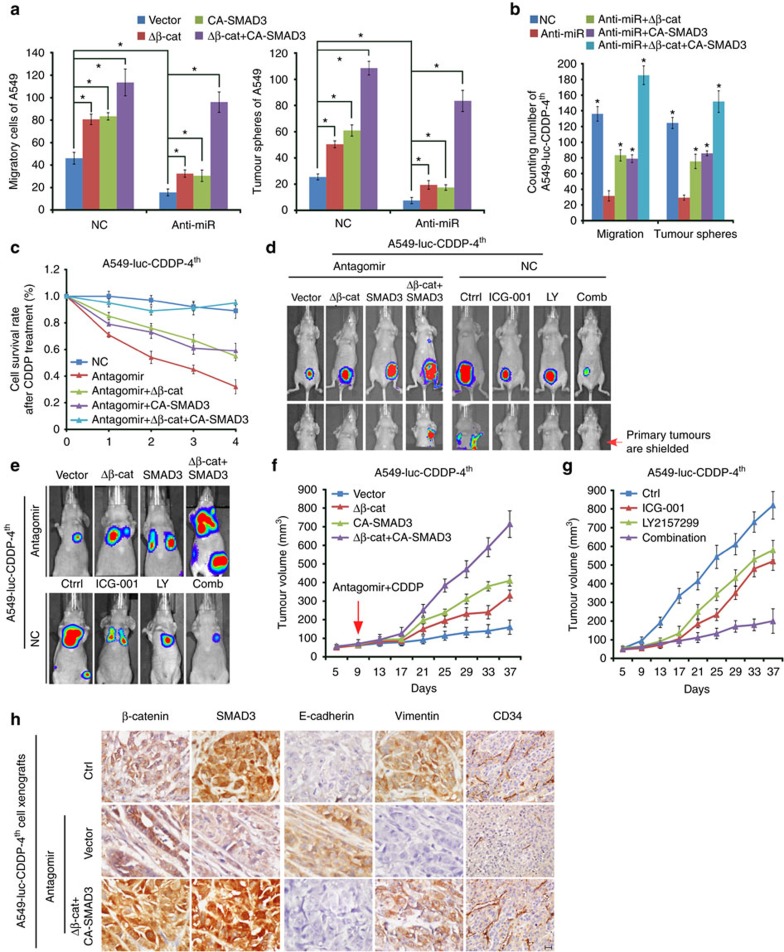
β-catenin and TGF-β signalling overactivation mediates miR-128-3p-induced aggressiveness in NSCLC cells. (**a** and **b**) Effects of introducing constitutively active β-catenin (Δβ-catenin) and SMAD3 (CA-SMAD3) mutants, alone or in combination, on cell migration and tumour cell sphere growth in A549-luc-CDDP-4th (A) and A549 (B) cells transfected with NC or miR-128-3p inhibitor (Anti-miR). (**c**) Cell viability assays determining the resistance or sensitivity of A549-luc-CDDP-4th cells treated as indicated to CDDP. (d and e) Mice bearing tumour xenografts by subcutaneous inoculation (**d**) or experimental metastasis by intravenous injection (**e**) of A549-luc-CDDP-4th cells transduced with the constitutively active β-catenin and SMAD3 mutants, alone or in combination, were treated by intravenous administration of miR-128-3p antagomir, or by intraperitoneal injection of with ICG-001 and LY2157299, alone or in combination (Comb). Bioluminescent images of subcutaneous tumours and spontaneous/experimental metastasis are shown. (**f**) Tumour growth curves of A549-luc-CDDP-4th cells with the indicated pretreatments in mice intravenously administered miR-128-3p antagomir and CDDP in combination. (**g**) Tumour growth curve of A549-luc-CDDP-4th cells in mice intraperitoneally injected with the indicated inhibitors. (**h**) Immunostaining of β-catenin, SMAD3, E-cadherin, Vimentin and CD34 in tumour tissues of the indicated A549-luc-CDDP-4th cell xenografts. Scale bar: 25 μm. Error bars represent mean±s.d. derived from three independent experiments. A two-tailed Student’s *t*-test was used for statistical analysis (*P<0.05).

Next we examined the importance of the β-catenin and TGF-β pathways in miR-128-3p-mediated effects. Constitutively active β-catenin and SMAD3 mutants, namely Δβ-catenin and CA-SMAD3 together not only completely reversed the inhibitory effects of miR-128-3p antagonism on the migration and self-renewal abilities of A549 cells (Fig. 6a) but also enabled A549-luc-CDDP-4th cells to fully resist miR-128-3p antagomir treatment or combined CDDP administration as evidenced by drastically increased cell survival, tumorigenesis and distant metastases of A549-luc-CDDP-4th cells (Fig. 6b–f and Supplementary Fig. 8b). By contrast, Δβ-catenin or CA-SMAD3 alone only partially restored the inhibitory effect of miR-128-3p antagonism on the tumour growth of A549-luc-CDDP4th cell xenografts whereas hardly restored the metastatic capacity of the primary tumours. On the other hand, inhibitors of β-catenin or TGF-β signalling, ICG-001 and LY2157299, respectively, when administered in combination, exhibited remarkable inhibitory effects, similar to that of miR-128-3p antagonism, on tumour growth and spontaneous/experimental metastasis of A549-luc-CDDP-4th cells, whereas ICG-001 or LY2157299 treatment alone also to certain degrees displayed therapeutic effects (Fig. 6d,e, g and Supplementary Fig. 8b). Furthermore, nuclear location of β-catenin and SMAD3, and weak E-cadherin, high Vimentin and igh CD34 staining were observed in miR-128-3p antagomir-, or CDDP-resistant xenografts formed by the A549-luc-CDDP-4th cells experimentally expressing Δβ-catenin and CA-SMAD mutants or A549-luc-miR-128-3p cells. In contrast, the corresponding derived xenografts sensitive to miR-128-3p antagomir treatment or its combination with CDDP, showed staining of the inactive membrane-associated β-catenin and cytoplasmic SMAD3, levels of high E-cadherin, low Vimentin and low CD34, and high proportion of apoptotic cells (Fig. 6h and Supplementary Fig. 8c). These data suggest specific and essential roles of both Wnt/β-catenin and TGF-β signalling in mediating the promoting effects of miR-128-3p in NSCLC.

### miR-128-3p targets Axin1, SFRP2, WIF1, SMURF2 and PP1c

**Figure 7:**
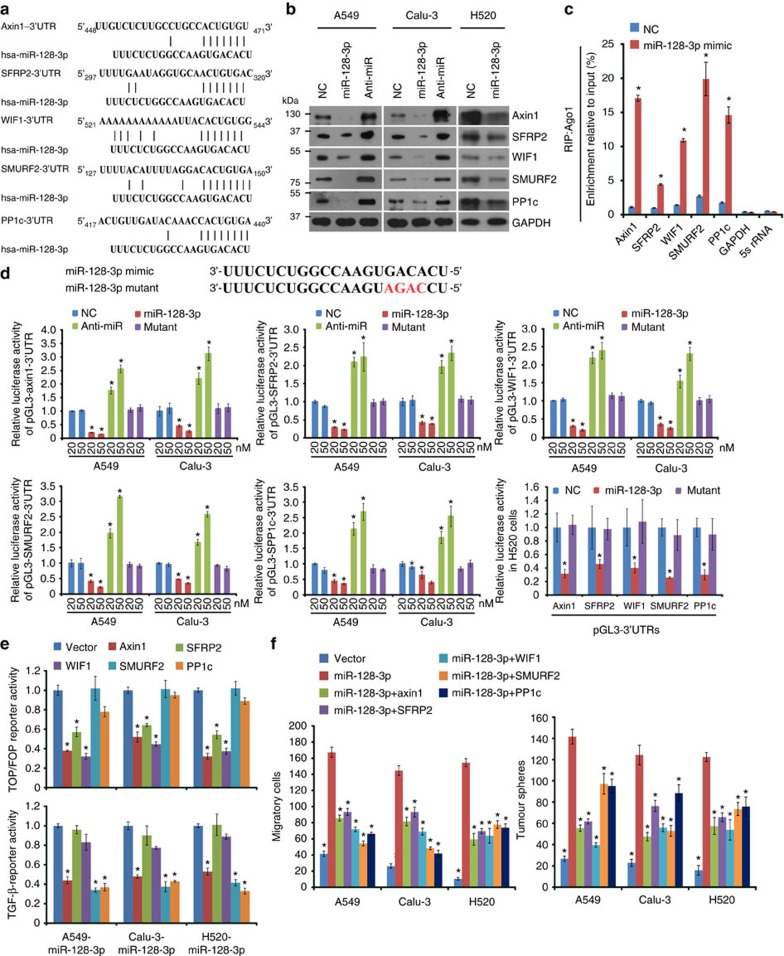
miR-128-3p targets Axin1, SFRP2, WIF1, SMURF2 and PP1c in NSCLC cells. (**a**) Targetscan tool showing schematic representation of putative binding sites for miR-128-3p in 3′-UTRs of Axin1, SFRP2, WIF1, SMURFS and PP1c. (**b**) WB analysis of the protein levels of Axin1, SFRP2, WIF1, SMURF2 and PP1c in the indicated cells. (**c**) By immunoprecipitation against Ago1, RIP analysis reveals the interaction of miR-128-3p with the 3′-UTRs of Axin1, SFRP2, WIF1, SMURF2 or PP1c mRNA to form miRNP complexes. IgG immunoprecipitation, as well as the interaction of miR-128-3p with GAPDH and 5s rRNA, were used as negative controls. (**d**) Luciferase assay of pGL3-Axin1-3′-UTR, pGL3-SFRP2-3′-UTR, pGL3-SMURF2-3′-UTR, pGL3-PP1c-3′-UTR or pGL3-WIF1-3′-UTR reporters in the indicated cells, co-transfected with increasing amounts (20 and 50 nM) of the indicated oligonucleotides. The sequence of the miR-128-3p mutant is shown. (**e**) Effects of restored expression of Axin1, SFRP2, WIF1, SMURFS and PP1c in miR-128-3p-overexpressing cells on luciferase activities of the TOP/FOP reporter and TGF-β reporter. (**f**) Effects of restored expression of miR-128-3p target genes on cell migration and self-renewal measured by Transwell migration assay and sphere formation assay, respectively, in the indicated NSCLC cells. Error bars represent mean±s.d. derived from three independent experiments. A two-tailed Student’s *t*-test was used for statistical analysis (*P<0.05, **P<0.01).

Three negative regulators of the β-catenin pathway, Axin1, SFRP2 and WIF1 and two negative mediators, SMURF2 and PP1c, of the TGF-β pathway^18^, predictively contain putative binding sites for miR-128-3p in their respective 3′-untranslated regions (UTRs; Fig. 7a). Protein levels, other than mRNA levels, of these five genes were remarkably reduced in NSCLC cells overexpressing miR-128-3p but were upregulated in the miR-128-3p-silenced NSCLC cells (Fig. 7b and Supplementary Fig. 9a). RNA-immunoprecipitation analysis showed that the transcripts of these five candidate target genes were indeed specifically assembled into the miR-128-3p mimic oligonucleotides-containing miRNPs (Fig. 7c). Moreover, luciferase activity of the reporter linked with the 3′-UTR of each target gene was dose-dependently repressed by miR-128-3p mimic transfection, but reversely increased following miR-128-3p inhibition and mutation of the miR-128-3p mimic completely abolished the repressive effects (Fig. 7d). Re-expressing Axin1, SFRP2 or WIF1 suppressed β-catenin signalling activity, and Axin1, SMURF2 or PP1c inhibited TGF-β signalling activity in miR-128-3p-overexpressing cells (Fig. 7e and Supplementary Fig. 9b). Furthermore, separately restoring ORFs of these target genes (without the 3′-UTR) partially abrogated the migratory and self-renewal abilities of miR-128-3p-transduced NSCLC cells (Fig. 7f). In parallel, silencing each target gene in miR-128-3p-silenced A549 cells partially reversed the inhibitory effect of miR-128-3p antagonism on the migratory and self-renewal abilities of NSCLC cells (Supplementary Fig. 9c,d), suggesting an importance of inhibiting each of the five target genes for miR-128-3p-induced aggressiveness.

**Figure 8:**
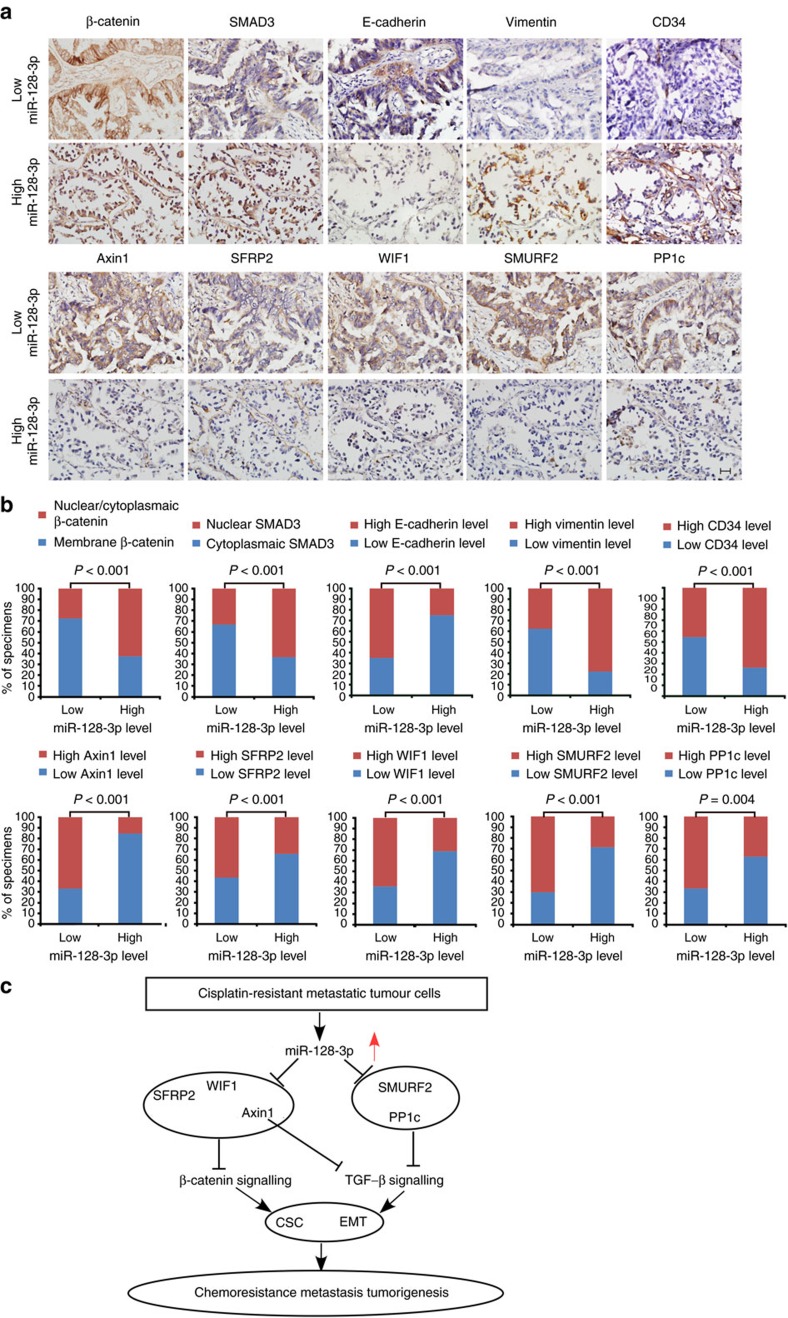
Clinical relevance of miR-128-3p with activation of β-catenin and TGF-β signalling, and expression levels of its target genes and pro-EMT/pro-metastatic markers. (**a**) miR-128-3p expression levels correlate with localization of β-catenin and SMAD3, as well as expression of its target genes, including Axin1, SFRP2, WIF1, SMURF2 and PP1c, two key EMT markers (E-cadherin and Vimentin) and the endothelial marker CD34. Two representative cases (Low and High miR-128-3p) are shown. Scale bar, 25 μm. (**b**) Percentage of specimens showing cytoplasmic/nuclear or membrane β-catenin, nuclear or cytoplasmic SMAD3, low- or high expression of E-cadherin, Vimentin, Axin1, SFRP2, WIF1, SMURF2 or PP1c and CD34 intensity in patient specimens, respectively, with low and high miR-128-3p expression. (**c**) Model for miR-128-3p-mediated tumorigenesis, metastasis and chemoresistance in NSCLC.

The tight association between high miR-128-3p expression and low levels of its five target proteins, as well as the consequent activation of β-catenin and TGF-β signalling, was evidenced in freshly resected NSCLC tissues (Supplementary Fig. 9e) and the cohort of 153 cases of paraffin-embedded human NSCLC specimens (Fig. 8a,b). Of note, 63.2% and 62.9% of specimens with high miR-128-3p expression exhibited nuclear/cytoplasmic-localized β-catenin and nuclear-localized SMAD3, respectively. In contrast, membrane β-catenin staining and inactive cytoplasmic SMAD3 staining were observed in 72.7% and 66.3% of specimens with low miR-128-3p expression, respectively. Moreover, in the high miR-128-3p expression group, 84.2%, 65.8%, 68.4%, 71.0% and 63.1% of samples, respectively, showed low levels of Axin1, SFRP2, WIF1, SMURF2 and PP1c, whereas these molecules were otherwise highly expressed in 67.5%, 57.1%, 64.9%, 70.1% and 66.2% of specimens expressing low miR-128-3p, respectively. In addition, 25.0%, 77.6% and 73.7% of NSCLC specimens with high miR-128-3p expression exhibited high levels of E-cadherin, Vimentin and endothelial marker CD34, respectively. By contrast, 35.1%, 62.3% and 54.5% of NSCLC specimens with low miR-128-3p expression expressed low levels of E-cadherin, Vimentin and CD34, respectively (Fig. 8a,b), suggesting tight clinical correlations among miR-128-3p levels, activated β-catenin and TGF-β signalling, and pro-EMT/pro-metastatic protein expression levels. Altogether, these results indicate that miR-128-3p-directed CSC and EMT programming contributes to tumorigenesis, metastasis and chemoresistance in NSCLC through concomitantly overactivating the β-catenin and TGF-β signalling (Fig. 8c).

## Discussion

Previously, models for chemoresistance and metastasis were largely investigated separately in most types of cancer, leaving common mechanisms underlying the development of chemoresistance and chemoresistance-associated metastasis poorly illustrated. Recently, Acharyya *et al*. uncovered a network of endothelial-carcinoma-myeloid signalling interactions linking chemoresistance and metastasis through the establishment of an interesting model showing that chemotherapy triggered TNF-α production by endothelial and other stromal cells and heightened CXCL1/2 expression via NF-κB activation in breast cancer cells, thus causing chemoresistance and metastasis^10^. In the present study, we established a progressive chemoresistant *in vivo* model of NSCLC simultaneously presenting spontaneous distant metastasis and mimicking concurrent chemoresistance and tumour cell dissemination observed in the clinical course of NSCLC. We further demonstrated the importance of intrinsic cellular programming of EMT and CSC in chemoresistance and metastasis, and provided a direct molecular link controlling EMT and CSC programming in NSCLC cells. This finding suggests that chemoresistance and metastasis can both be due to cell-intrinsic programming in NSCLC, in addition to the host environment-tumour interaction observed in breast cancer^10^. In addition, together with Acharyya’s findings and other previous observations that treatment with chemotherapeutic drugs such as cisplatin or paclitaxel, adversely enhanced pulmonary metastases^19^,^20^, our study suggests that although chemotherapy alone might give rise to transient inhibition of primary tumour growth, the combination of chemotherapy with therapies targeting CSC formation might be of greater therapeutic value in overcoming chemoresistance and metastasis.

Our chemoresistance-associated metastasis model of NSCLC xenograft, together with functional and clinical studies, highlights a determinant role of miR-128-3p in chemoresistance and metastasis in the cancer type. On the backdrop of this notion though, interestingly, numerous previous reports claimed contradictory expression status and/or biological effects of miR-128 in different cancer types and even within one cancer type^21^,^22^,^23^,^24^,^25^,^26^,^27^,^28^,^29^,^30^. Indeed, we found that miR-128-3p overexpression potentiated chemoresistance in several cell lines of colon cancer, pancreas cancer and hepatocellular carcinoma, whereas miR-128-3p overexpression potentiated chemosensitivity in breast cancer and oesophageal cancer cells lines, and overexpressing miR-128-3p displayed opposite effects on chemoresistance in different glioma cell lines (data not shown). While such discrepancies remain to be clarified by more future independent investigations, it is noteworthy that some of these previous studies showed downregulation of miR-128-3p in cancer cells or tissues, including NSCLC^30^, which contradicts against the data in the TCGA and other public data sets, as well as against the data presented by a systemic analysis of miRNA profiling in multiple types of cancers^15^. Our current study employed an A549-derived chemoresistance/metastasis model and demonstrated that miR-128-3p is among the most markedly upregulated miRNAs in the chemoresistant, metastatic A549-luc-CDDP-4th cells, in agreement with the results obtained from analysing several independent large public lung cancer databases. Consistent with our results, another previous report described that miR-128-3p could be upregulated by a direct transactivation of oncogenic mutant p53 (R175H) and conferred increased resistance to cisplatin, doxorubicin and 5-fluorouracyl treatments in NSCLC cells^31^. Clinically, high miR-128-3p expression levels in NSCLC tissues correlated with poor prognosis, disease progression and poor curative responsiveness in two independent large NSCLC patient cohorts. More importantly, antagonizing miR-128-3p in various highly malignant lung cancer cell lines, not only abrogated tumorigenesis, primary tumour growth and distant metastasis, but also re-sensitized them to chemotherapeutic drugs, which further can be completely rescued by restored activation of the Wnt/β-catenin and TGF-β signalling, suggesting that in NSCLC miR-128-3p acts as a tumour-promoting molecule that promotes cancer progression, especially in chemoresistance and metastasis. It is also important to note that although we observed the oncogenic role of miR-128-3p in most NSCLC cell lines, several NSCLC cell lines seem to be irresponsive to either miR-128-3p overexpression or its inhibition (data not shown), indicating a heterogeneous response even in a same cancer type. Therefore, it will be of great interest to further identify NSCLC patient subpopulations whose tumours are sensitive to miR-128-3p antagonism. In addition, since results obtained from cell lines may not ideally reflect clinical reality, larger cohorts of clinical NSCLC specimens are still needed to validate the biological significance and clinical implication of miR-128-3p.

CSCs with metastatic potential are increasingly becoming attractive targets for developing strategies to address drug resistance and metastatic relapse. However, these approaches under investigation still face remarkable challenges, largely due to the apparent lack of useful stem cell-specific markers to identify CSCs^32^. Although the cell-surface phenotype has been used to enrich CSCs, mounting evidence shows that CSCs undergo dynamic changes in the levels of cell-surface markers, making them inconsistent, and thus, it is difficult to therapeutically target the CSC subpopulation based on these membrane markers^33^,^34^. In addition, Curtis *et al*.^35^ characterized phenotypic varieties of lung tumour-propagating cells with distinct genetic alterations of oncogenes/tumour suppressors and suggested that it would be difficult to effectively restrict CSCs through targeting characterized mutant oncogenes/tumour suppressors. On the other hand, identifying modulatory networks of the signalling pathways underlying the development and presentation of CSC properties might provide promising opportunities for designing effective treatment regimens^32^,^34^. Among such signalling pathways, the Wnt/β-catenin and TGF-β pathways have been highlighted as critical players in the generation and maintenance of CSCs as well as in the development of EMT, and these pathways contribute tremendously to tumour relapse, metastasis and chemoresistance^14^. In response to this understanding, investigative agents targeting metastatic CSCs via inhibiting the β-catenin or TGF-β pathways are being developed. For example, ICG-001 analogues, which specifically antagonizes the CBP/β-catenin interaction to eliminate drug-resistant CSCs in several tumour types, are in Phase I clinical trial^36^, and LY2157299, a small molecule inhibitor of the TGF-β receptor, is being investigated in Phase I/II studies for metastatic malignancies^37^,^38^. The effectiveness of the drug category can be compromised by their disruption of only a small subset of targeted protein interactions or by the limited inhibition on a single pathway^36^,^38^,^39^, and their long-term effects remain unclear^38^,^40^. Here, our work shows that a treatment regimen with ICG-001 and LY2157299 in combination remarkably suppressed tumorigenesis and distant metastasis of chemoresistant, metastatic NSCLC cells as effectively as miR-128-3p antagonism, thus highlighting the significance of Wnt/β-catenin and TGF-β signalling activated by miR-128-3p upregulation in NSCLC in inducing CSC and EMT programming and consequently chemoresistance-associated metastasis.

Although deregulated activation of both β-catenin and TGF-β signalling cascades have been implicated in NSCLC, the molecular basis for such deregulation remains puzzling as genetic mutations of core components in the pathways are rare, unlike in other cancer types^41^,^42^. Our finding that upregulated miR-128-3p in NSCLC cells simultaneously suppresses multiple negative regulators of β-catenin and TGF-β signalling, including Axin1, SFRP2, WIF1, SMURF2 and PP1c, provides unique insights in understanding the modulation of the β-catenin and TGF-β pathways in lung cancer. Consistent with our findings, decreased Axin expression has previously been found to correlate with poor differentiation and progression in NSCLC, and Axin overexpression inhibits proliferation and invasion of NSCLC cell lines^43^,^44^. In a similar context, SFRP2 and WIF1, whose expression levels are frequently silenced in NSCLC, suppress growth and induce chemosensitivity of tumour cells^45^,^46^,^47^,^48^. Moreover, SMURF2 knockout mice are prone to developing a variety of types of cancers including lung cancer; therefore, SMURF2 has been identified as a bona fide tumour suppressor^49^. In addition, PP1c could function as an important kinase inhibitor of the TGF-β pathway and block tumorigenesis and migration^50^. These previous findings have highlighted the importance of a regulatory network of β-catenin and TGF-β signalling in the course of NSCLC development and progression. Therefore, our current finding that miR-128-3p could confer simultaneous suppression upon multiple inhibitors of β-catenin and TGF-β signalling has underscored the key roles of these miR-128-3p-targeted inhibitors in providing fine-tuned control of β-catenin and TGF-β signals in NSCLC. In summary, using a chemoresistance-associated metastasis experimental model and *in vivo* as well as *in vitro* functional and molecular assays, in combination with extensive clinical correlation analyses, our study provides a basis for developing strategies to abrogate the β-catenin and TGF-β together by antagonizing one molecule in NSCLC so as to simultaneously target chemoresistance and metastasis.

## Methods

### Cell culture

NSCLC cell lines, including A549, Calu-3, SK-MES-1, PAa, NCI-H292, NCI-H520 (H520), NCI-H596, NCI-H1299, NCI-H1650, NCI-H1975, HLAMP and 95D were obtained from cell banks of Shanghai Institutes of Biological Sciences (Shanghai, China) or Fu Erbo Biotechnology Co., Ltd. (Guangzhou, China). Murine lung carcinoma cell line LL/2-luc-M38 was provided by Caliper Life Sciences, Inc (Massachusetts, USA). Subcutaneous tumours-derived cells were primarily cultured and primary normal lung epithelial cells were obtained instructed by the protocol of previous report^51^. NSCLC cell lines were maintained in DMEM medium (Invitrogen) supplemented with 10% fetal bovine serum (HyClone, Logan, UT) and 1% penicillin/streptomycin (Invitrogen). The BEAS-2B immortalized human bronchial epithelial cell line (Shanghai Institutes of Biological Sciences, Shanghai, China) was cultured in LHC-9 medium as instructed by the provider. All cell lines were authenticated by short tandem repeat fingerprinting at IDEXX RADIL (Columbia, MO, USA) and Services at SYSU Forensic Medicine Lab (Guangzhou, China), and were free of mycoplasma contamination.

### Tissue specimens

Clinical NSCLC specimens conducted in this study were obtained from, and histopathologically diagnosed at, the Sun Yat-sen University Cancer Center. Clinicopathological staging of the tumour samples were determined and characterized according to the current International Union Against Cancer Tumour–Node–Metastasis classification. For the use of these clinical materials for research purposes, prior patients’ consents and approval from the Institutional Research Ethics Committee were obtained. Clinical information of a cohort of 153 cases of NSCLC specimens are presented in Supplementary Fig. 2a. In another independent cohort of 234 cases of NSCLC specimens receiving multiple rounds of CDDP-based first-line therapy, except 26 patients with unknown therapeutic effect, curative responsiveness for NSCLC patients, including PR (at least a 30% decrease in the sum of diameters of target lesions, taking as reference the baseline sum diameters, *n*=64), SD (at least a 20% increase in the sum of diameters of target lesions and an absolute increase of at least 5 mm in the sum, taking as reference the smallest sum on study or the appearance of one or more new lesions, *n*=122) or PD (neither shrunk sufficiently to qualify for PR nor increased sufficiently to qualify for PD, taking as reference the smallest sum diameters while on study, *n*=22), was evaluated by clinical oncologists according to the Response Evaluation Criteria in Solid Tumours version 1.1 (ref. 52).

### Plasmid construction and viral transduction

Identical mature miR-128 transcripts can be encoded by two distinct genes, MIR128-1 and MIR128-2, which are embedded in the introns of the R3HDM1 gene on chromosome 2q21.3 and the ARPP21 gene on chromosome 3p22, respectively. Of particular note, in addition to encoding identical mature sequences of miR-128-3p species, the MIR128-1 and MIR128-2 genes also encode miR-128-1-5p and miR-128-2-5p species, respectively, which differ slightly in sequences. Interestingly, the absolute amounts of miR-128-1-5p and miR-128-2-5p species are far lower than those of miR-128-3p species, as evidenced in the miRbase database. To achieve overexpression of miR-128-3p, a DNA fragment containing the hsa-miR-128-2 precursor with 300 bp genomic sequences flanking each side was amplified into the retroviral plasmid pMSCV-puro (Clontech, Palo Alto, CA). Constitutively active β-catenin and SMAD3 mutants, namely, Δβ-catenin (90 amino acids in the N-terminal domain deleted^53^) and CA-SMAD3 (mutation to aspartic acid of the three C-terminal serine residues in SMAD3 (ref. 54)) constructs, were subcloned into pMSCV-puro and pMSCV-hygro vector plasmids, respectively. The resultant plasmids were individually co-transfected with the pIK packaging plasmid in 293FT cells by using a standard calcium phosphate transfection method as previously described^55^. After 36 h, supernatants were collected and filtered to infect A549 and Calu-3 cells for 24 h in the presence of polybrene (2.5 μg ml^−1^). Puromycin or hygromycin was used to select cells stably overexpressing miR-128-3p, Δβ-catenin or CA-SMAD3 for 10 days. The open reading frames (ORFs) of Axin1, SFRP2, WIF1, SMURF2 and PP1c generated by PCR amplification were separately cloned into the mammalian expression vector pcDNA3.1 (Invitrogen) and their 3′-UTRs were separately amplified into the downstream of the luciferase gene in a pGL3 control vector (Promega, Madison, WI). TOP and FOP reporters containing wild-type and mutated TCF/LEF DNA binding sites, respectively, were purchased from Upstate Biotechnology (Lake Placid, NY), and the TGF-β- (p3TP-lux), the STAT3-, Hedgehog- and NF-κB-responsive reporters were obtained from Addgene Inc (Cambridge, MA).

### Western blotting and immunofluorescence assays

Western blotting analyses were performed using anti-E-cadherin (BD562869), anti-γ-catenin (BD 610253), anti-Vimentin (BD550513) and anti-N-cadherin (BD561553) (BD Biosciences, San Diego, CA); anti-cleaved Caspase 3 (#9661), anti-PARP (#9542), anti-CCND1 (#2922), anti-Axin1 (#2087), anti-LEF1 (#2230), anti-phosphor-SMAD3 (ser423/425) (#9520), and anti-non-phosphor- (Ser33/37/Thr41)-β-catenin (#8814) (Cell Signaling, Danvers, MA); anti-CTR2 (ab58777), anti-ABCG2 (ab108312), anti-SFRP2 (ab137560), anti-WIF1 (ab186845), anti-PP1c (ab134947), anti-SMURF2 (ab191697), anti-SMAD3 (ab40854), anti-phosphor-SMAD3 (ab52903), and anti-TCF4 (ab185736) (Abcam, Burlingame, CA). anti-GAPDH (ab28925, abcam) or anti-β–actin (#4970, Cell Signaling) was used as a loading control. Relative protein levels were quantified by scanning densitometry, and the relative grey value of protein was calculated as Band Intensity of Protein of Interest/Band Intensity of Loading Control for analysing their association with increasing folds of miR-128-3p expression. Immunofluorescence staining assay was carried out using anti-E-cadherin, anti-γ-catenin, anti-Vimentin, anti-N-cadherin, anti–β-catenin (BD610153, BD Biosciences), anti-SMAD2/3 (ab207447, abcam) and Rhodamine-conjugated goat anti-mouse secondary antibody (Thermo Fisher, Waltham, MA). Uncropped scans of important blots are provided as Supplementary Fig. 10 in the Supplementary Information.

### RNA extraction and quantitative real-time PCR

Total miRNAs of cultured cells and surgically resected NSCLC tissues were extracted using the mirVana miRNA Isolation Kit (Ambion, Austin, TX) according to the manufacturer’s instructions. Total miRNAs of paraffin-embedded, archived clinical NSCLC specimens were extracted using the RecoverAll Total Nucleic Acid Isolation Kit (Ambion) according to the manufacturer’s instructions. miR-128-3p expression was quantified by real-time PCR using the miRNA-specific TaqMan MiRNA Assay Kit (Applied Biosystems, Foster City, CA), which was assessed based on the threshold cycle (Ct) value and calculated as 2^−[(Ct of miR-128-3p) – (Ct of U6)]^ with the expression level of U6 small nuclear RNA as the normalization reference. The copy number of miR-128-3p or miR-128-2-5p was calculated by absolute quantitation according to a previous report^56^,^57^. In detail, starting with a 1 nM solution of an high-performance liquid chromatography (HPLC) -purified synthetic oligoribonucleotide standard (Invotrogen) identical in sequence to hsa-miR-128-3p or or hsa-miR-128-2-5p, a primary dilution of 32.8 pM was created by adding 4 μl of the 1 nM solution to 118.1 μl H_2_O, mixed and centrifuged. From this primary dilution, ten serial 4-fold dilutions were made by adding 20 μl of the previous solution to 60 μl RNase-free, DNase-free H_2_O, such that the eleventh tube contains ∼10 copies of miR-128-3p or miR-128-2-5p mimetic per pg small RNA based on the average Molecular Weight (MD) of a 21nt mature miRNA. The twelfth tube containing H_2_O only was used as a no-template control. Plotting Ct values versus copy number of the synthetic miR-128-3p or miR-128-2-5p in a standard curve allowed fitting of a curve that was then used to approximate copies of miR-128-3p or miR-128-2-5p from Ct values obtained with biological samples. Among the 12 tumour samples tested for Fig. 1f, two NSCLC specimens were randomly picked for each of four NSCLC stages, i.e., stages I, II, III and IV, respectively, from our cohort of 153 cases of NSCLC specimens, and additional two Stage IV (PD) specimens were randomly picked from our another independent cohort of 234 NSCLC patients having received multiple rounds of CDDP-based first-line therapy, and the other two non-cancerous lung tissues were adjacent normal tissues correspondingly paired with the stage II NSCLC specimens. Total mRNAs were extracted using Trizol (Invitrogen) and their expression levels were normalized to GAPDH and analysed using Gene Expression Assays (Promega).

### Cell invasion and wound healing assays

Transwell migration (without matrigel) assay and Matrigel invasion assay were performed as previously described^58^. At 24∼36 h after cell plating, invading cells were fixed, stained, photographed and quantified by counting them in five random 200x magnification fields. For three-dimensional invasive culture, indicated cells (5 × 10^3^) were trypsinized, mixed with 10% Matrigel and seeded on Matrigel coated in 24-well plates. Culture medium was refreshed every other day and formed three-dimension spheroid structures were photographed under 200x magnification every 3 days for 12 days. For wound healing assay, after 24 h starvation for cell cycle synchronization, confluent monolayer of cells, seeded in six-well plates, were scratched with sterile 200-μl pipette tips for artificially creating wounds. The wound healing process was observed and photographed under × 100 magnification at indicated time points.

### Tumour sphere culture

Indicated cells (2.5 × 10^3^), seeded in ultra-low adherent six-well plates (Corning, Cambridge, MA), were cultured in DMEM/F12 serum-free medium (Invitrogen) supplemented with 2% of B27, 20 ng ml^−1^ of EGF (BD Biosciences), 20 ng ml^−1^ of bFGF and 4 mg ml^−1^ insulin (Sigma) to form tumour spheres. Nutrient supplemented medium was added for the growth of spheres every 2 days for 10 days. To further evaluate the self-renewal potential of these cells, spheres were collected by gentle centrifugation, dispersed into single cells, and re-cultured as described above for three generations, and were photographed under × 200 magnification, or harvested for RNA isolation.

### Flow cytometry analysis

For CD133 immunofluorescent staining, single cell suspension was prepared, labelled with phycoerythrin-conjugated CD133 antibody (Miltenyi Biotec, Cologne, Germany) according to the manual instruction and analysed using a Beckman Coulter EPICS XL flow cytometer (Fullerton, CA). Side population (SP) analysis was performed using flow cytometer BD influx (BD Biosciences) with standard protocol^59^, and Verapamil (50 μmol l^−1^) was added to inhibit ABCG2 transporter and to confirm the SP. SP and non-SP cells were sorted according to Verapamil gating for isolation of total miRNA.

### RNA immunoprecipitation

Immunoprecipitation of miRNP with anti-Ago1 (Abcam, Cambridge, MA) or anti-IgG (Sigma) was performed as previously described^60^. In brief, cells were lysed in buffer containing 100 mM KCl, 5 mM MgCl_2_, 10 mM HEPES (pH7.4) and 0.5% NP-40; and the immune complex captured by protein A agarose was washed in buffer containing 150 mM KCl, 5 mM MgCl_2_, 10 mM HEPES (pH7.4) and 0.1% NP-40 for 6 times. RNA extraction was performed using RNAeasy Kit (Qiagen, Valencia, CA).

### Transfection and luciferase reporter assays

Oligonucleotides including miR-128-3p mimics, anti-miR-128-3p (Anti-miR) and corresponding vector controls, and siRNAs against CTR1, CCND1, JAK2, STAT3, GLI, P65, Axin1, SFRP2, WIF1, SMURF2, PP1c, TCF4, LEF1, SMAD3 and SMAD4 were purchased from Invitrogen. Transfection of plasmids or oligonucleotides was performed using Lipofectamine 2000 reagent (Invitrogen) for functional assays, luciferase reporter assays or protein and RNA analyses. For the luciferase reporter assays, cells with 70% confluence in 24-well plates were transfected with indicated luciferase reporters (200 ng) and 1 ng of pRL-TK Renilla luciferase construct. Forty-eight hours after transfection, cell extracts were subjected to measurement by Dual Luciferase Reporter Assay Kit (Promega) according to the manufacturer’s instruction.

### miRNA sponge

The miR-128-3p sponge, a construct carrying six tandem repeats of miR-128-3p binding sites contained in the 3′-UTR of a GFP expression cassette, was constructed using a modified method based on previous reports^61^. Briefly, synthetic oligonucleotides containing six repeated miR-128-3p binding sites with BamHI and SpeI digestion sites were annealed and ligated into the 3′-UTR of EGFP mRNA in the lentiviral plasmid pSin-EF2-EGFP, which was subcloned from the pSin-EF2-Sox2-puro plasmid (Addgene) digested with EcoRI and SpeI to substitute EGFP for Sox2. Control sponge containing irrelevant sequences was correspondingly established.

### Cell viability assay and chemotherapeutics drugs treatment

Cell viability was evaluated using the 3-(4,5-Dimethyl-2-thiazolyl)-2,5-diphenyl-2H-tetrazolium bromide (MTT) assay. A total of 1 × 10^3^ cells were seeded in 96-well plates, and chemotherapeutic drugs including CDDP (Sigma), gemcitabine (Lilly France, Fegersheim, France) and paclitaxel (Sigma) with indicated final concentrations were separately added for 2–4 days. After treatment with chemotherapeutic drugs, the incubation media were removed, and the cells were stained with 100 μl sterile MTT dye (0.5 mg ml^−1^, Sigma, Saint Louis, MO) for 4 h at 37 °C, followed by removal of the culture medium with 150 μl of dimethyl sulphoxide (DMSO) (Sigma). After shaking at room temperature for 10 min, the absorbance of stained cells was measured at 570 nm, with 655 nm as the reference wavelength. Cell survival rate was calculated as a percentage of corresponding control solvent.

### TUNEL assay

Terminal deoxynucleotidyl transferase-mediated dUTP-biotin nick end labelling (TUNEL) assay in cultured cells was performed according to our previous report^62^. Apoptotic cells in paraffin-embedded tissue sections were determined by using the TUNEL assay (Roche Diagnostics, Indianapolis, IN) according to the manufacturer’s instructions. Briefly, after deparaffinization and rehydration, tissue sections were digested with proteinase K for 20 min at 37 °C. Slides were rinsed with PBS and incubated with 3% H_2_O_2_ in PBS for 10 min to block endogenous peroxidase activity, followed by washing with 0.02% Tween 20 in PBS on ice. Sections were then pre-incubated in TdT Reaction Buffer for 10 min and incubated in TdT Reaction Mixture for 1.5 h at 37 °C in a humidified chamber, followed by washing with Stop Wash Buffer for 10 min to stop reaction. After an additional wash with 0.02% Tween 20 in PBS, sections were incubated with peroxidase-conjugated streptavidin in a humidified chamber for 30 min at room temperature, incubated in DAB solution for 2 min and counterstained with hematoxylin for 30 s. After washing in running tap water and dehydration in ethanol, slides with coverslip could be observed under microscopy. The presence of nuclear brown staining was indicative of apoptotic cells.

### Anchorage-independent growth assay

A total of 1 × 10^3^ cells were trypsinized and suspended in 2 ml complete culture medium containing 0.3% agar (Sigma). The agar-cell mixture was grown on the top of 1% agar contained in complete medium. An amount of 500 μl culture medium containing 3 μg CDDP or control solvent was refreshed every 3 days. After 12 days, viable cellular colonies larger than 25 μm in diameter were counted and photographed under × 200 magnification.

### Uptake analysis of chemotherapeutic drugs

Uptake analysis of CDDP, gemcitabine and paclitaxel was determined by flame atomic absorption spectroscopy and HPLC/tandem mass spectrometry, respectively, according to previous reports^63^,^64^. Indicated cells with 80% confluency were incubated, respectively, with CDDP (3 μg ml^−1^) for 30 min, gemcitabine (0.5 μg ml^−1^) for 3 h or paclitaxel (0.5 μg ml^−1^) for 3 h, refreshed with regular medium for 0, 60, 120, 180 and 240 min and then harvested for centrifugation. Each cell pellet at the above time points was washed twice with ice-cold PBS and divided into two equal aliquots. One of the aliquots was subject to protein quantification by Bradford method, and the other alioquot was determined for drug concentration. The intracellular drug quantity was expressed as pg of CDDP, gemcitabine or paclitaxel per mg of protein. For CDDP concentration determination, cell pellets were digested overnight at 60 °C with 1 M benzethonium hydroxide (Sigma), followed by acidification with 1 N HCl, and the CDDP amount was determined by flame atomic absorption spectroscopy. For measurement of gemcitabine and paclitaxel levels, cell pellets were ultrafiltered, derivatized and extracted with 200 μl chloroform. Finally, 50 μl of the chloroform layer was analysed on an HPLC/tandem mass spectrometry system for detecting gemcitabine or paclitaxel concentrations.

### Immunohistochemistry

IHC assays using anti-β-catenin, anti-SMAD3, anti-Axin1, anti-SFRP2, anti-WIF1, anti-SMURF2, anti-PP1c, anti-E-cadherin, anti-Vimentin or anti-CD34 were separately conducted on paraffin-embedded specimens of 153 cases of NSCLC patients. The degree of immunostaining of indicated proteins was scored by the staining index (SI), which was calculated as staining intensity × percentage of positive tumour cells. The staining intensity was quantified as: 0 (no staining); 1 (weak staining=light yellow); 2 (moderate staining=yellow brown); and 3 (strong staining=brown), and percentage of positive tumour cells was scored as: 0 (no positive tumour cells); 1 (<10%), 2 (10–50%); and 3 (>50%). β-catenin was defined as positive when located in cytoplasm or nuclear, and negative with distributed membrane staining^65^. The optimal cutoff or high- and low-expression level of protein of interest was identified as: the SI score ≥4 as high expression, and ≤3 as low expression.

### Xenograft tumour model and drug administration *in vivo*

For bioluminescent assays, A549 cells were stably transduced with pMSCV-luciferase-neo plasmid, followed by transduction of pMSCV-pri-miR-128-2-puro or empty vector to generate A549-luc-miR-128-3p or A549-luc-Vector cells, and an identical level of luciferase expression was verified in these two engineered cell lines. To establish a chemoresistant subcutaneous tumour model, after inoculation of A549-luc-Vector cells, cisplatin (*cis*-diaminedichloroplatinum, CDDP, 4 mg kg^−1^) (Sigma) or control solvent was intraperitoneally (i.p.) injected six times within 2 weeks for a round of treatment. To assure that the experimental mice survive repeatedly administered CDDP treatments long enough to be able to present with chemoresistance and metastasis and to validate the drug sensitivity *in vitro*, as well as to obtain chemoresistant, metastatic NSCLC cells, cells were isolated from the corresponding resultant tumours, cultured in petri dishes for approximately 3 weeks (1 week in neomycin-contained medium to eliminate non-tumour cells and approximately 2 additional weeks in regular culture medium without addition of CDDP in order to alternatively mimic the break between chemotherapy courses in the clinic) and re-transplanted into syngeneic mice for receiving the next round of CDDP or control treatment until the occurrence of chemoresistant tumours. For the tumorigenicity model, indicated cells of five dosages (5 × 10^6^, 5 × 10^5^, 5 × 10^4^, 5 × 10^3^ and 5 × 10^2^) were subcutaneously inoculated into different female BALB/c nude mice (6–7 weeks of age, 20–24 g). Primary tumours were shielded to better visualize distant organ metastases by avoiding the interference of the overwhelming bioluminescent intensities exerted by the primary tumour. For the experimental metastasis model, the indicated cells (2 × 10^6^) were intravenously (i.v.) injected, and metastases were monitored every 3 days. Bioluminescent imaging was performed using the Xenogen IVIS Spectrum (Caliper Life Sciences). Fifteen minutes before imaging, the mice were injected i.p. with 150 mg kg^−1^ luciferin. Following anaesthesia, live images were capture by photography. At the experimental end points, systemic organs were immediately resected for *ex vivo* evidence of metastatic signals, and then haematoxylin and eosin staining was then performed for histological confirmation of metastasizing tumour cells, followed by immunostaining using antibodies for MUC1, CK5 and CD34 (abcam, Cambridge, MA). For *in vivo* antagomir administration, after the indicated cells were subcutaneously xenografted (5 × 10^6^ for A549-luc-CDDP-4th, 5 × 10^5^ for A549-luc-miR-128-3p, 1 × 10^6^ for LL/2-luc-M38) or intravenously injected (2 × 10^6^ for all), 50 μl miR-128-3p antagomir or control antagomir (diluted in PBS at 2 mg ml^−1^) was i.v. administered six times within 2 weeks. Combination treatment with 2 mg ml^−1^ miR-128-3p antagomir (i.v.) plus 2 mg kg^−1^ CDDP (i.p.) for 6 consecutive days was performed, and a single injection of 4 mg kg^−1^ CDDP six times within 2 weeks was performed. Two inhibitors, namely, ICG-001 (2 mg kg^−1^) and LY2157299 (2 mg kg^−1^), were intraperitoneally injected alone or in combination. At least five nude mice per group were used to ensure the adequate power and each mouse with different weight was randomly allocated. Bioluminescent imaging of primary tumours and metastases was not performed in a blinded manner. All animal studies were approved by the Sun Yat-sen University Institutional Animal Care and Use Committee.

### miRNA array and bioinformatic analysis

Total RNA samples from A549-luc-CDDP-4th and control cells were extracted for microRNA microarray analysis, and those from A549-miR-128-3p and A549-Vector cells were collected for mRNA microarray analysis. The microarray experiments were carried out commercially by the Shanghai Biochip Corporation following standard Agilent protocols. Bioinformatics analysis and visual heatmaps were performed with the MeV 4.4 program (http://www.tm4.org/mev/). Genespring GX 11 software package (Agilent, Santa Clara, CA) was utilized to identify statistically significant pathways. Microarray data have been deposited in the National Center for Biotechnology Information Gene Expression Omnibus (http://www.ncbi.nlm.nih.gov/geo/), with accession number GSE67729, including the miRNA expression profiling under accession number GSE67727 and mRNA expression profiling under accession number GSE67728.

### Statistics

Two independent cohorts of NSCLC patients were respectively divided into two groups based on miR-128-3p expression level, namely, the low-miR-128-3p expression group (below or equal to the median value) and the high-miR-128-3p expression group (above the median value), for clinical survival analysis. All statistical analyses were performed with the SPSS 13.0 statistical software package following the REMARK reporting guidelines. The correlation between miR-128-3p expression and clinicopathological characteristics was analysed by the χ^2^-test. The survival curve was established by the Kaplan–Meier method and compared by the log-rank test. Cox-regression models were performed for univariate and multivariate analysis. Student’s *t*-test (two-tailed) was used to compare statistical significance between two groups. Sample size was determined by power analysis to achieve a minimum effect size of 0.5 with a *P* value of <0.05 and all sample sizes were appropriate for assumption of normal distribution. Variance within each group of data was estimated and was similar between compared groups. Data analysis was performed by two independent investigators who were blinded to the sample groups. *P*<0.05 was considered statistically significant in all cases.

### Data availability

The authors declare that the data supporting the findings of this study are available within the article, its Supplementary Information files or from the authors.

## Additional information

**How to cite this article:** Cai, J. *et al*. Simultaneous overactivation of Wnt/β-catenin and TGFβ signalling by miR-128-3p confers chemoresistance-associated metastasis in NSCLC. *Nat. Commun.* **8**, 15870 doi: 10.1038/ncomms15870 (2017).

**Publisher’s note:** Springer Nature remains neutral with regard to jurisdictional claims in published maps and institutional affiliations.

## Supplementary Material

Supplementary Information
