# Publisher Correction: Simultaneous overactivation of Wnt/β-catenin and TGFβ signalling by miR-128-3p confers chemoresistance-associated metastasis in NSCLC

**DOI:** 10.1038/ncomms16196

**Published:** 2018-03-30

**Authors:** Junchao Cai, Lishan Fang, Yongbo Huang, Rong Li, Xiaonan Xu, Zhihuang Hu, Le Zhang, Yi Yang, Xun Zhu, Heng Zhang, Jueheng Wu, Yan Huang, Jun Li, Musheng Zeng, Erwei Song, Yukai He, Li Zhang, Mengfeng Li

Nature Communications 8: Article number: 15870; DOI: 10.1038/ncomms15870 (2017); Published online 06 19 2017; Updated 03 30 2018

In the original version of this Article, the affiliation details for Yan Huang and Li Zhang were incorrectly given as ‘Department of Medical Oncology, Sun Yat-Sen University Cancer Center, Guangzhou 510060, China’, and should have been ‘State Key Laboratory of Oncology in South China, Department of Medical Oncology, Sun Yat-Sen University Cancer Center, Guangzhou 510060, China’. This has now been corrected in both the PDF and HTML versions of the Article.

