# Supplementary material for: Simultaneous overactivation of Wnt/β-catenin and TGFβ signalling by miR-128-3p confers chemoresistance-associated metastasis in NSCLC
Source: Nat Commun. 2017 Jun 19;8:15870. doi: 10.1038/ncomms15870 (PMC5481840; doi:10.1038/ncomms15870)

Type of file: PDF
Size of file: 0 KB
Title of file for HTML: Supplementary Information
Description: Supplementary Figures

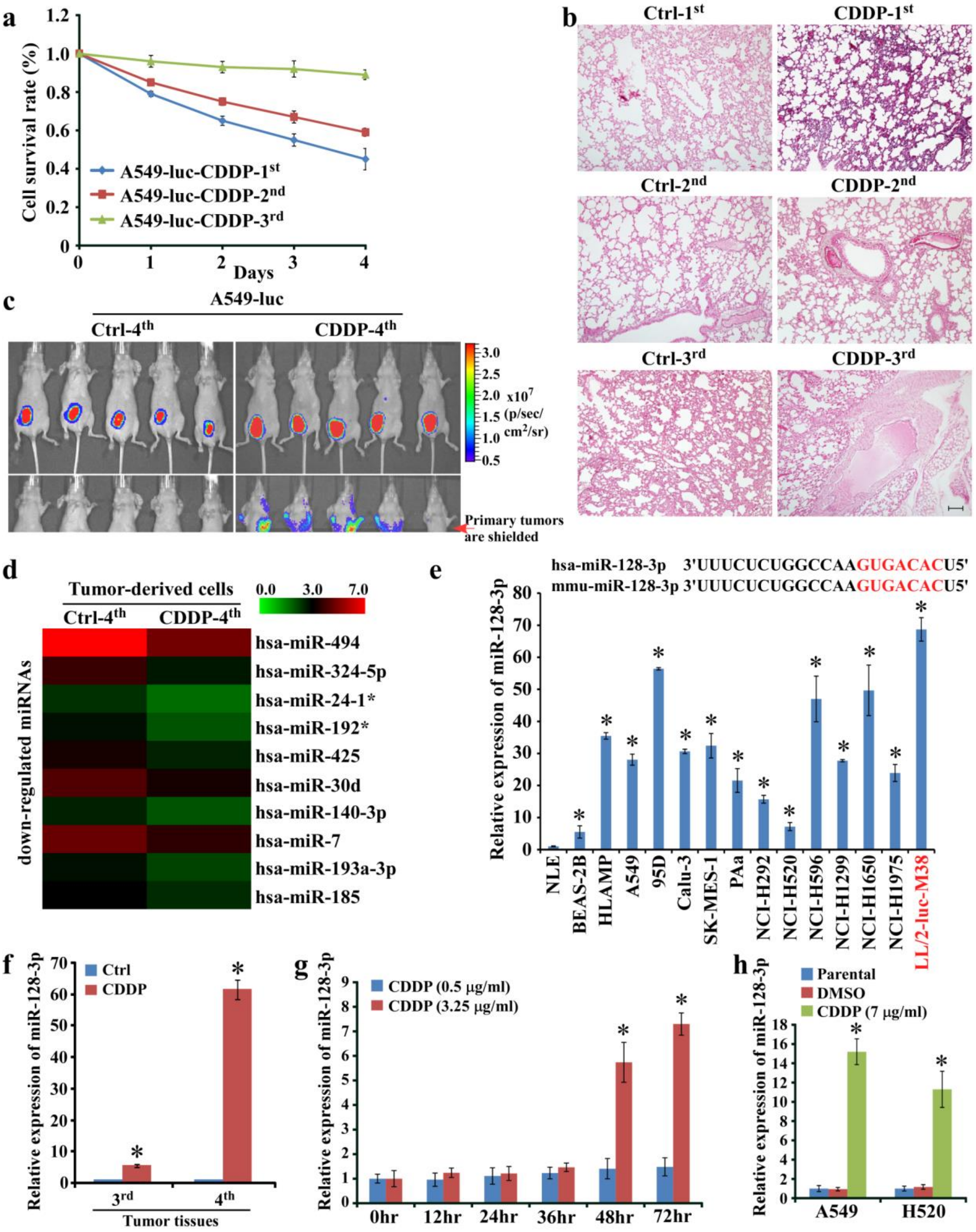

Supplementary Figure 1 miR-128-3p is highly expressed in chemoresistant, metastatic NSCLC xenografts, NSCLC cell lines and CDDP-resistant NSCLC cells. (a) Cell viability assays determining the resistance or sensitivity to CDDP (3μg/ml) treatment of indicated A549-luc cells obtained from resultant subcutaneous tumors surviving indicated rounds of CDDP

treatment. (b) Lung tissues obtained from mice bearing NSCLC xenografts at the indicated rounds of Ctrl and CDDP treatment were histologically examined. Original magnification, x400. (c) Bioluminescent images of subcutaneous tumors inoculated with indicated cells in response to CDDP-4th and Ctrl-4th treatment. To observe spontaneous metastasis, primary tumors were shielded. (d) Tumor-derived cultured cells from Ctrl-4th and CDDP-4th treatment were subjected to miRNA array analysis, revealing the top 10 downregulated miRNAs in chemoresistant, metastatic A549-luc-CDDP-4th cells. Intensity values representing miRNA expression levels were $\log_{10}$ transformed. (e) qRT-PCR analysis of relative miR-128-3p expression in a panel of NSCLC cell lines and a naturally highly metastatic murine lung cancer cell line LL/2-luc-M38 compared to primary normal lung epithelial cells (NLE) and immortalized human bronchial epithelial cell line BEAS-2B. (f) QRT-PCR analysis of miR-128-3p expression in tumor tissues obtained from mice at the indicated rounds of CDDP treatment. (g) qRT-PCR analysis of miR-128-3p expression in A549 cells treated at low dose (0.5μg/ml) or IC_{50} concentration (3.25 μg/ml) at the indicated time points. (h) qRT-PCR analysis of miR-128-3p expression in A549 and H520 cells at the indicated treatments. A two-tailed Student's *t*-test was used for statistical analysis (* $P<0.05$).

a

Characteristics		miR-128-3p Low	miR-128-3p High	*P*-value
Agender	male	57	57	0.890
	female	20	19	
Age (y)	< = 50	24	22	0.764
	> 50	53	54	
Pathology	Squamous cell carcinoma	31	23	0.069
	Adenocarcinoma	24	37	
	Others	22	16	
Clinical stage	I	62	0	< 0.001
	II	14	15	
	III	1	46	
	IV	0	15	
T classification	T1	17	5	< 0.001
	T2	53	32	
	T3	7	29	
	T4	0	10	
N classification	N0	69	15	< 0.001
	N1	7	28	
	N2	1	32	
	N3	0	1	
Distant metastasis	No	77	61	< 0.001
	Yes	0	15	

d

	Univariate analysis			Multivariate analysis		
	P	HR	95% Confidence interval	*P*	HR	95% Confidence interval
T classification<br>T1<br>T2<br>T3<br>T4	0.001	1.518	1.183-1.947	0.538	1.089	0.830-1.428
N classification<br>N0<br>N1<br>N2<br>N3	< 0.001	1.911	1.503-2.431	0.285	1.204	0.857-1.692
Distant metastasis<br>No<br>Yes	0.015	2.108	1.143-3.888	0.956	1.018	0.536-1.934
miR-128-3p expression<br>Low<br>High	< 0.001	4.165	2.619-6.622	0.001	3.150	1.597-6.211

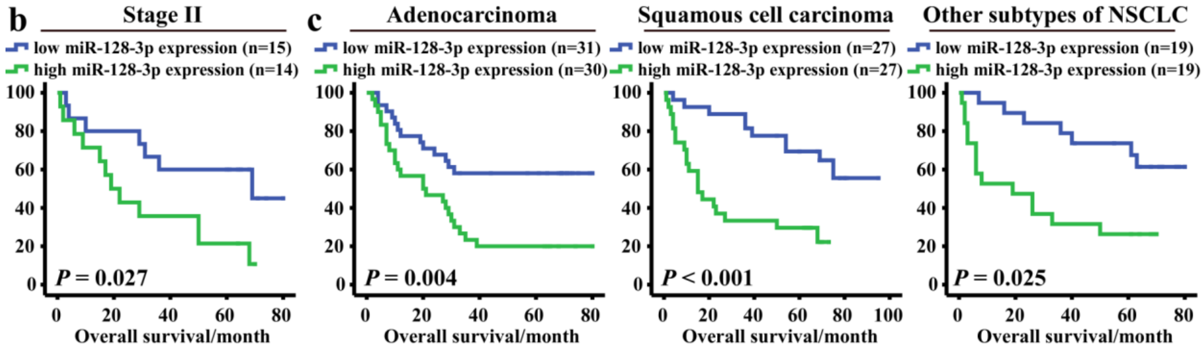

Supplementary Figure 2 Clinical relevance of miR-128-3p expression in NSCLC tissues. (a) Correlations between the clinicopathologic features and expression of miR-128-3p levels. (b) Kaplan-Meier analysis of overall survival of NSCLC patients at stage II, who were divided into the low-miR-128-3p expression group (below or equal to the median value) and the high-miR-128-3p expression group (above the median value). (c) Kaplan-Meier analysis of overall survival between the low and high miR-128-3p expression groups of a cohort of 153 NSCLC patients, who was further divided into distinct pathological subgroups, namely, adenocarcinoma, squamous cell carcinoma and other subtypes of NSCLC. (d) Univariate and multivariate analyses of various prognostic variables in a cohort of 153 NSCLC patients.

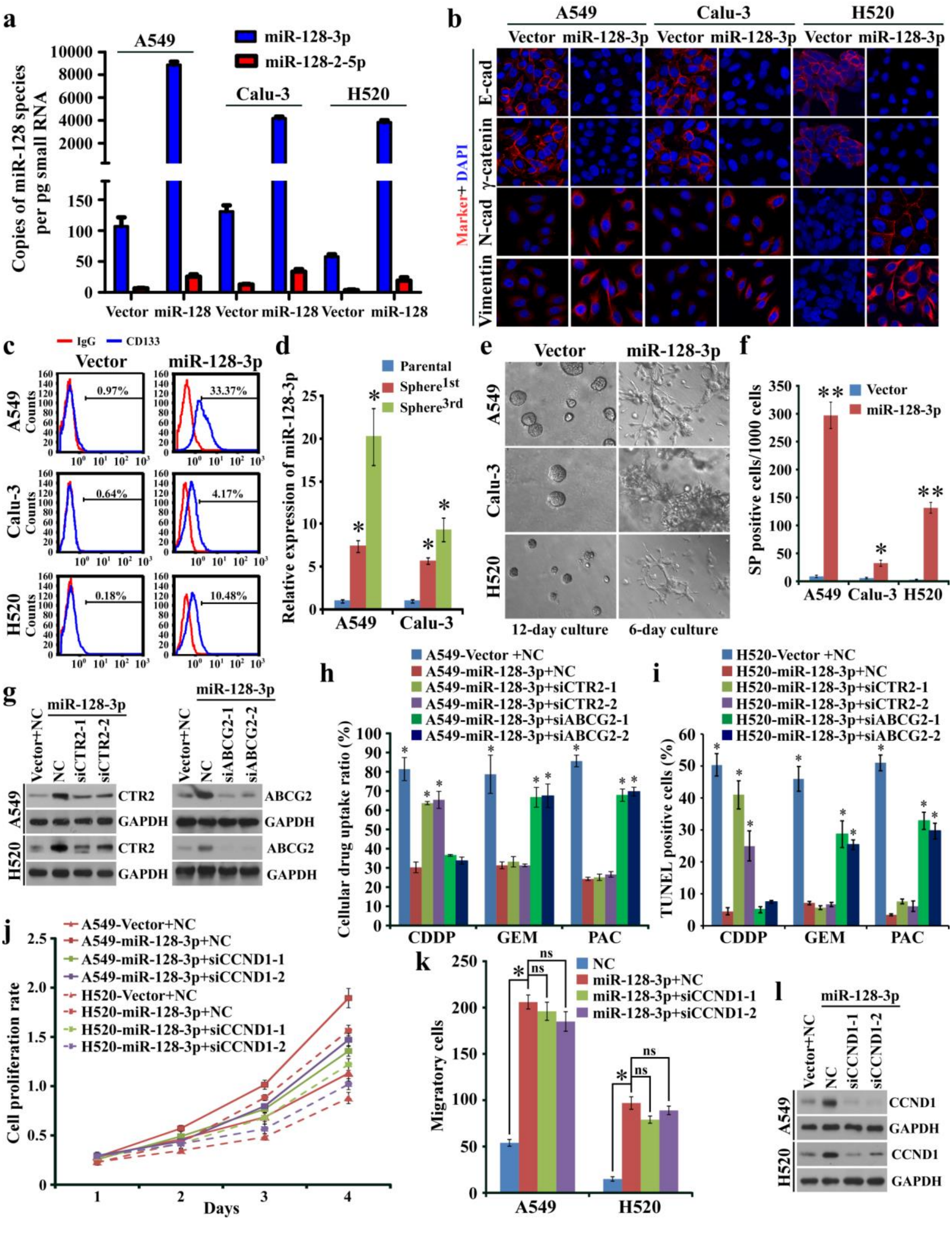

Supplementary Figure 3 Relevance of miR-128-3p expression in NSCLC cells with CSC and EMT characteristics. (a) Absolute real-time PCR using a standard curve of miR-128-3p or miR-128-2-5p expression in indicated cell lines. (b) Immunofluorescent staining of epithelial markers (E-cadherin and γ-catenin) and mesenchymal cell markers (N-cadherin and Vimentin).

(c) Flow cytometry was used to identify the CD133^{+} subpopulation of the indicated cells. (d) qRT-PCR analysis of miR-128-3p expression in parental NSCLC cells and cells derived from 3 passages of cultured tumor spheres. (e) Representative micrographs of the indicated cells grown on the Matrigel in 3-D invasive culture assay. (f) Flow cytometry analysis of the proportions of the side population (SP) for the indicated cells. Verapamil treatment was used as the negative control. (g) WB analysis of the expression of CTR2 and ABCG2 upregulated by miR-128-3p and the knockdown efficiency of indicated siRNAs in indicated miR-128-3p-overexpressing cells. (h) Kinetics of uptake of CDDP, gemcitabine (GEM) and paclitaxel (PAC) were determined by flame atomic absorption spectroscopy and high-performance liquid chromatography/tandem mass spectrometry (HPLC/MS/MS), respectively, in indicated cells. The cellular drug uptake ratio was calculated by the percentage of intracellular remaining CDDP content. (i) Quantification of TUNEL staining in indicated cells after CDDP (3μg/ml) gemcitabine (GEM, 0.5μg/ml), and paclitaxel (PAC, 0.5μg/ml) treatment, respectively. The numbers of TUNEL-positive cells were counted from 10 random fields and presented as percentages of total cell numbers. (j) MTT assay revealed cell growth curves of indicated cells. (k) Quantification of cell number of indicated migratory cells. (l) WB analysis of the expression of Cyclin D1 (CCND1) upregulated by miR-128-3p and the knockdown efficiency of siRNAs against CCND1 in indicated miR-128-3p-overexpressing cells. Error bars represent the means of three independent experiments. A two-tailed Student's *t*-test was used for statistical analysis (* $P<0.05$, ** $P<0.01$). Original magnification, b, x630; f and i, x200.

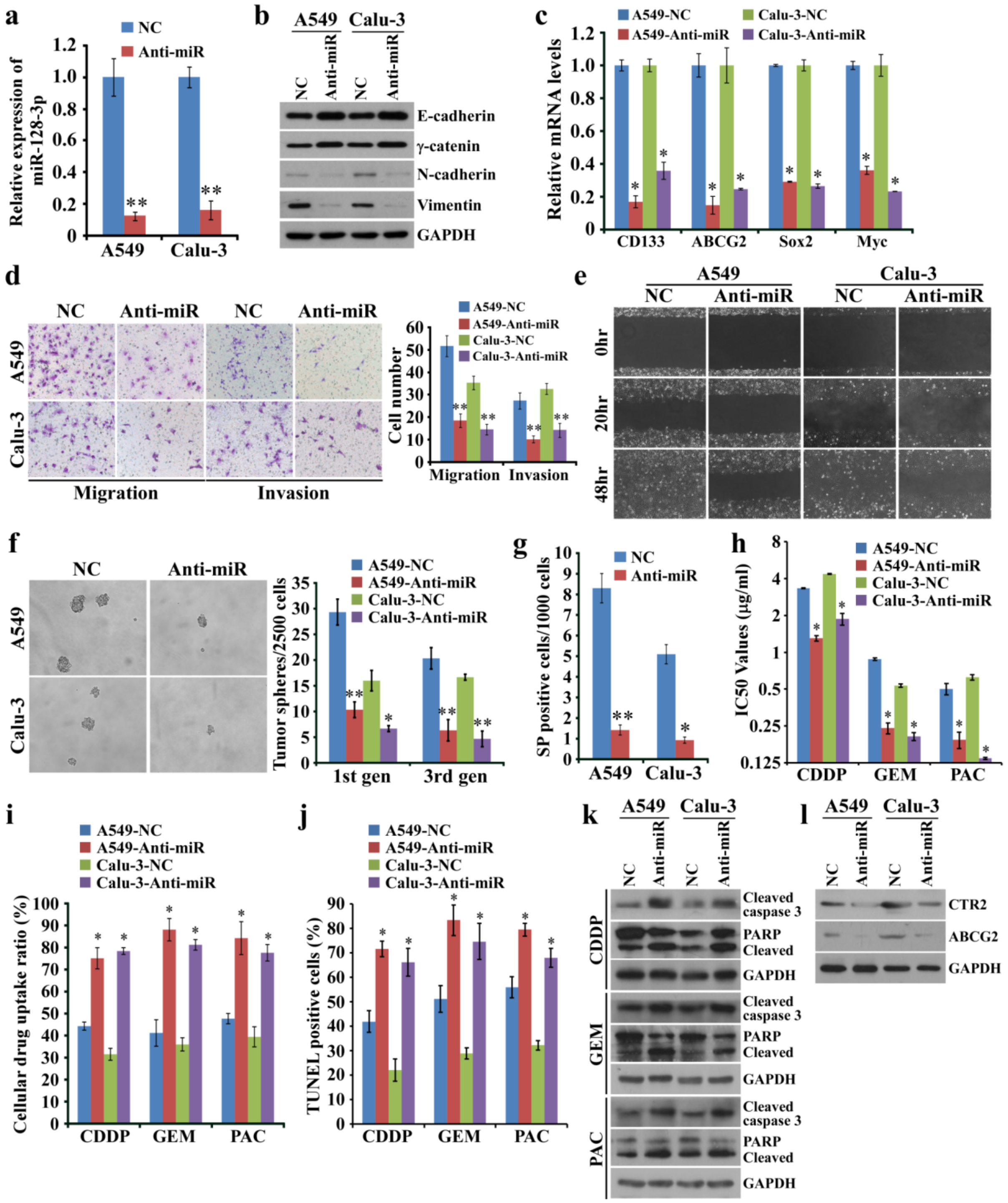

Supplementary Figure 4 Silencing endogenous miR-128-3p inhibits migration, invasion and self-renewal and promotes CDDP-induced apoptosis of NSCLC cells *in vitro*. (a) qRT-PCR analysis of miR-128-3p expression in the indicated cells transfected with anti-miR-128-3p (Anti-miR) or NC oligonucleotides. Transcript levels were normalized to *U6* expression. (b) WB analysis of epithelial and mesenchymal cell markers in cells transfected with

anti-miR-128-3p (Anti-miR) or control (NC) oligonucleotides. (c) Relative expression of embryonic and somatic stem cell markers related to cancer stemness, including Sox2, Myc, CD133 and ABCG2 in the indicated cells. (d) Representative images and average cell number of invading or migratory cells in five random fields examined by Matrigel-coated or -non-coated Transwell assays. (e) Representative micrographs of migration process of the indicated cells analyzed by wound healing assays. (f) Representative images (left) and quantification (right) of tumor spheres during 3 generations cultured from the indicated cells. (g) Flow cytometry analysis of the proportions of the side population (SP) for the indicated cells. Verapamil treatment was used as the negative control. (h) Cell viability assay of the indicated cells treated with CDDP, gemcitabine (GEM) and paclitaxel (PAC), respectively, at various concentrations to measure their respective IC50 values. (i) Kinetics of uptake of CDDP, gemcitabine (GEM) and paclitaxel (PAC) were determined by flame atomic absorption spectroscopy and HPLC/MS/MS, respectively, in indicated cells. The cellular drug uptake ratio was calculated by the percentage of intracellular remaining CDDP content. (j) Quantification of TUNEL staining in indicated cells after CDDP (3μg/ml) gemcitabine (GEM, 0.5μg/ml), and paclitaxel (PAC, 0.5μg/ml) treatment, respectively. The numbers of TUNEL-positive cells were counted from 10 random fields and presented as percentages of total cell numbers. (k) WB analysis for proteolytic cleavage of Caspase-3 and PARP in indicated cells after indicated treatment. (l) WB analysis of expression of CTR2 and ABCG2 in indicated cells. Error bars represent the means of three independent experiments. A two-tailed Student's *t*-test was used for statistical analysis (* $P<0.05$, ** $P<0.01$). Original magnification, d-f and k, x200.

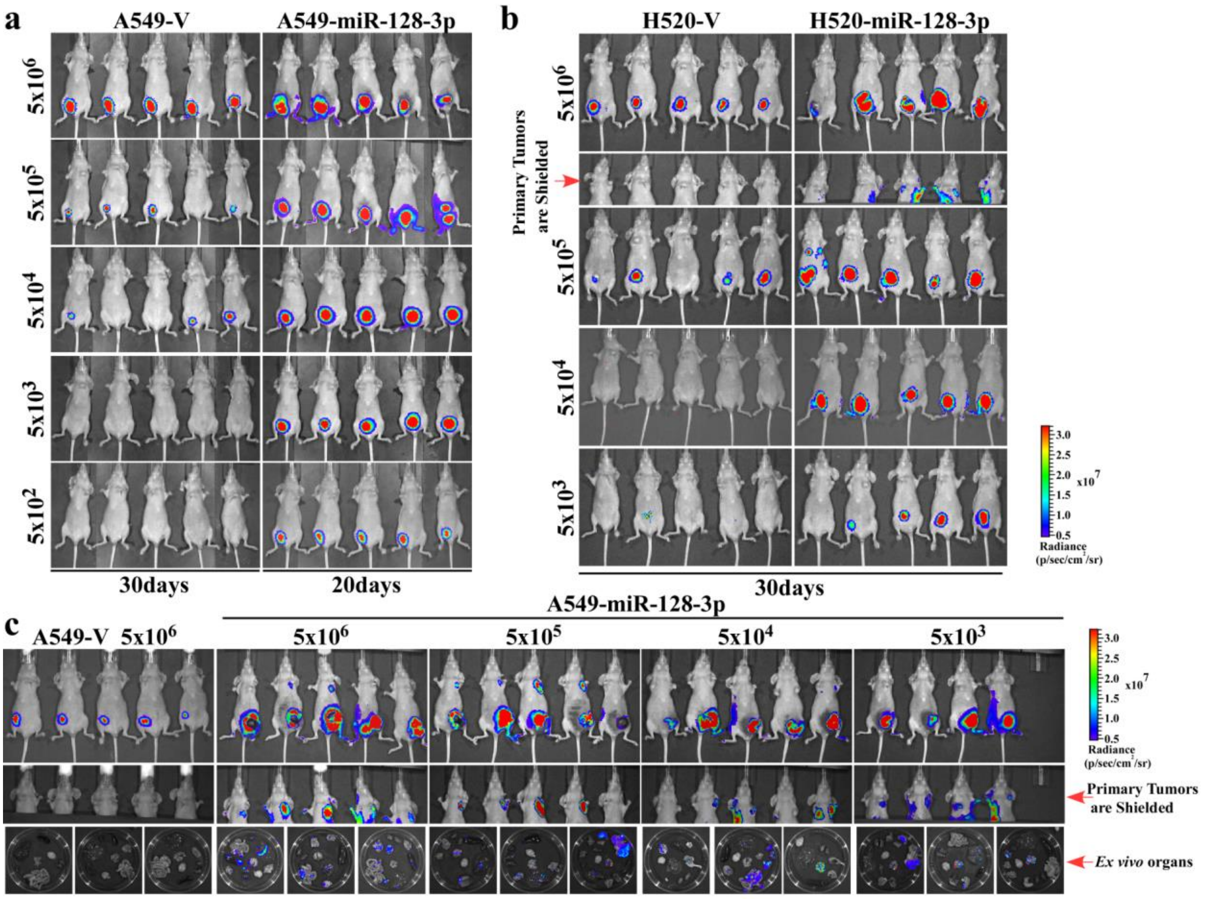

d

Number of tumor cells inoculated	Tumors incidence/Number of Injections					Metastatic tumors incidence /Number of Injections			
	$5x10^6$	$5x10^5$	$5x10^4$	$5x10^3$	$5x10^2$	$5x10^6$	$5x10^5$	$5x10^4$	$5x10^3$
A549-Vector	5/5	4/5	3/5	0/5	0/5	0/5	0/5	0/5	0/5
A549-miR-128-3p	5/5	5/5	5/5	5/5	5/5	5/5	5/5	4/5	4/5
H520-Vector	5/5	4/5	1/5	0/5	0/5	0/5	0/5	0/5	0/5
H520-miR-128-3p	5/5	5/5	5/5	4/5	0/5	4/5	2/5	0/5	0/5

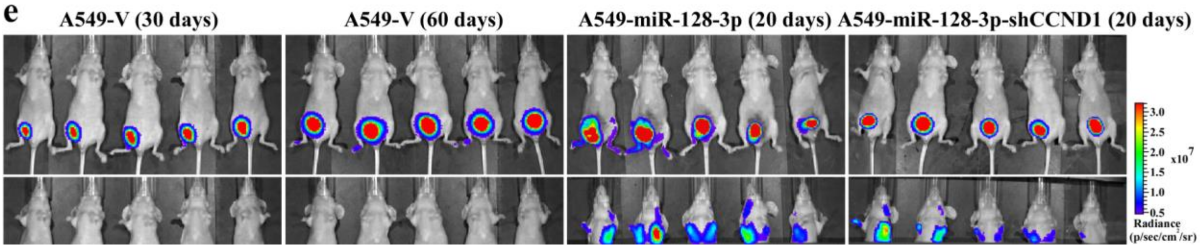

Supplementary Figure 5 miR-128-3p overexpression promotes tumorigenesis and spontaneous metastasis *in vivo*. (a) Bioluminescent images of each group of mice (n = 5/group) bearing subcutaneous tumors of A549-Vector (A549-V) and A549-miR-128-3p cells with the indicated cell densities are shown. (b and c) Bioluminescent images of subcutaneous tumors and

spontaneous metastasis of H520-Vector (H520-V) and H520-miR-128-3p cells (b) or A549-Vector and A549-miR-128-3p cells (c) with the indicated cell densities are shown. *Ex vivo* organ metastases confirmed the results. (d) Effect of miR-128-3p on the ability of NSCLC cells to develop measurable tumors or spontaneous metastasis. (e) Bioluminescent images of subcutaneous tumors and spontaneous metastasis from xenografts of A549-Vecotr, A549-miR-128-3p and CCND1-silenced A549-miR-128-3p (A549-miR-128-3p-shCCND1) cells at indicated time points.

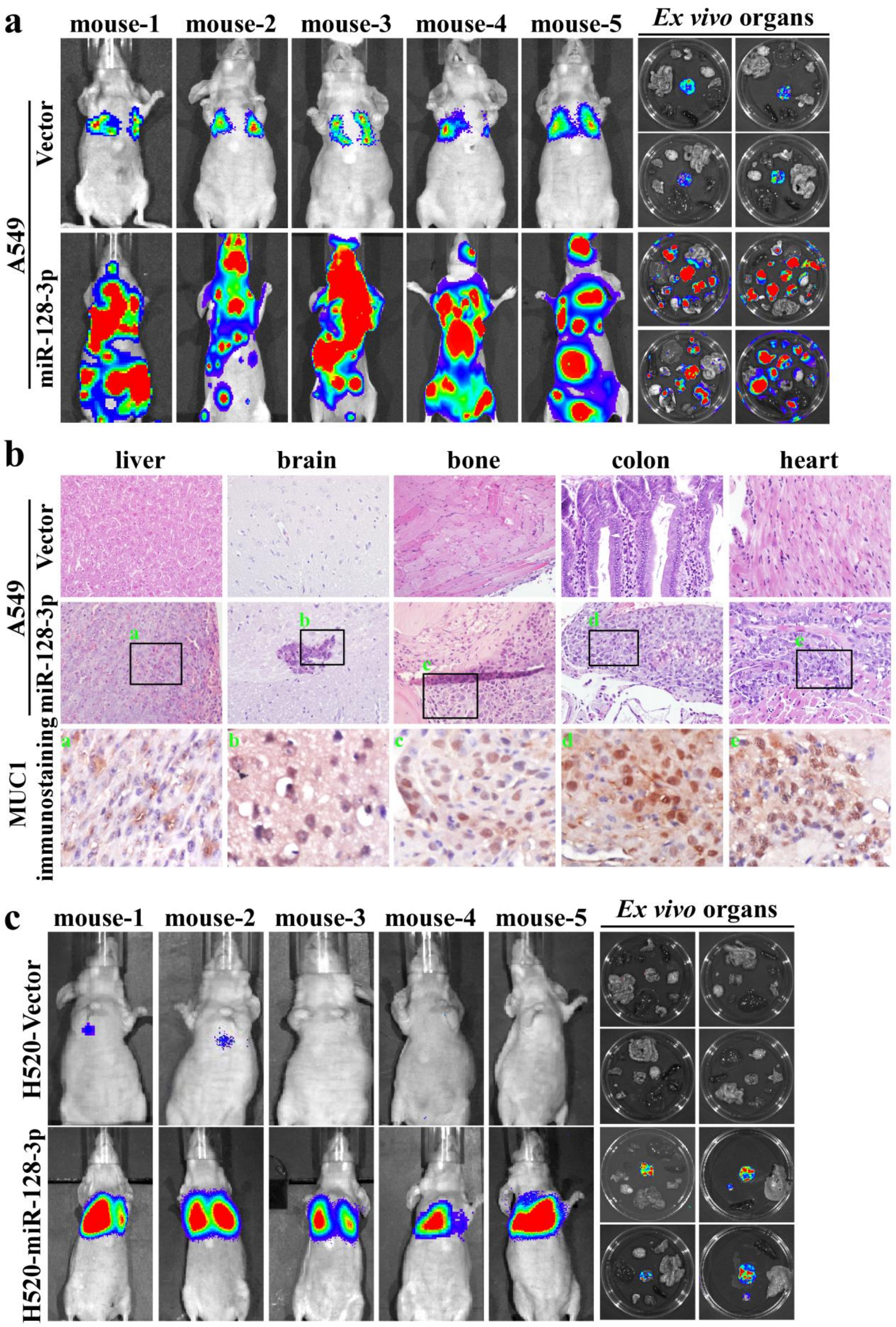

d

Inoculated Cells ($2x10^6$)	Metastases incidence/Number of Injections							
	lung	Liver	brain	spleen	kidney	colon	stomach	bones
**A549-Vector**	5/5	0/5	0/5	0/5	0/5	0/5	0/5	0/5
**A549-miR-128-3p**	5/5	5/5	5/5	5/5	5/5	5/5	5/5	5/5
**H520-Vector**	0/5	0/5	0/5	0/5	0/5	0/5	0/5	0/5
**H520-miR-128-3p**	5/5	0/5	0/5	0/5	0/5	0/5	0/5	0/5

Supplementary Figure 6 miR-128-3p overexpression promotes experimental distant metastasis

in vivo. (a-c) For experimental metastasis model, bioluminescent images of each group of mice (n = 5/group) bearing systemic metastases of the indicated cells are shown. *Ex vivo* organ metastases including lung, liver, spleen, kidney, colon, heart, stomach, bones and brain, confirmed the results (a and c). Representative organ metastases of A549-miR-128-3p cells were histologically confirmed by H&E staining and immunostaining of the lung adenocarcinoma marker MUC1 (b). (d) Effect of miR-128-3p on the ability of NSCLC cells to develop experimental distant organ metastases. Original magnification, b, x400.

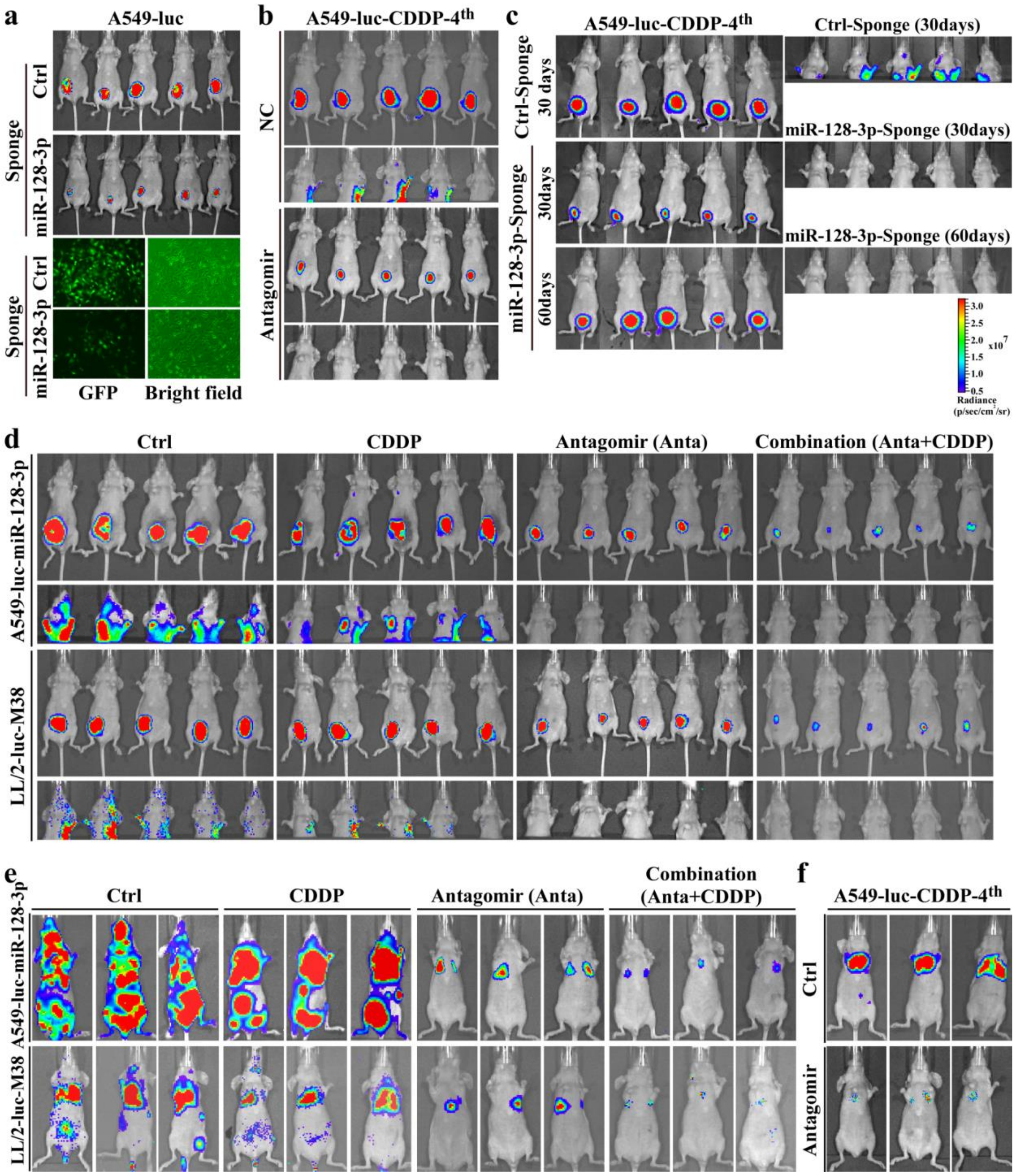

Supplementary Figure 7 Therapeutic effect of inhibiting miR-128-3p on tumor growth and distant metastasis and chemoresistance. (a) Bioluminescent images of each group of mice (n = 5/group) bearing subcutaneous tumors inoculated with A549-luc cells stably transduced with GFP-labeled control or miR-128-3p sponge (upper panel). Bright-field imaging and fluorescent visualization confirmed efficient inhibition of miR-128-3p by miRNA sponge strategy (lower panel). Original magnification, x200. (b) Bioluminescent images of each group of mice (n =

5/group) bearing subcutaneous tumors inoculated with A549-luc-CDDP-4th cells, with or without spontaneous metastasis (with tumors shielded) in response to intravenous administration of NC or miR-128-3p antagomir, respectively. (c) Bioluminescent images of each group of mice (n = 5/group) bearing subcutaneous tumors inoculated with A549-luc-CDDP-4th cells stably transduced with GFP-labeled control or miR-128-3p sponge at indicated experimental time points. (d) Bioluminescent images of each group of mice (n = 5/group) bearing subcutaneous tumors and spontaneous metastasis inoculated with A549-luc-miR-128-3p or LL/2-luc-M38 cells in response to the indicated treatments. (e and f) Bioluminescent images of each group of mice (n = 5/group) bearing experimental distant metastasis of the indicated cells in response to the indicated treatments.

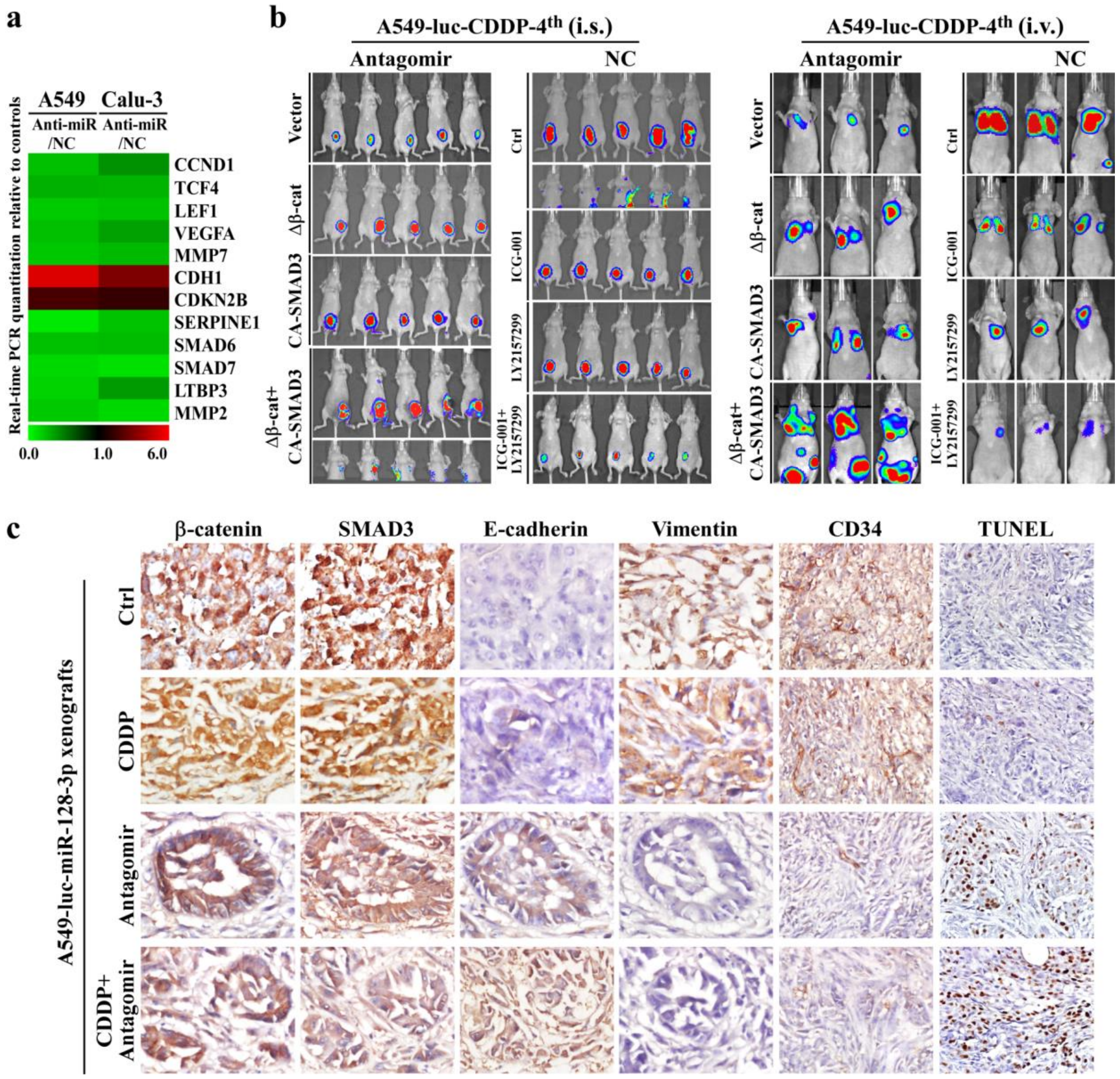

Supplementary Figure 8 The β-catenin and TGF-β signaling activation essentially mediates miR-128-3p-induced tumorigenesis and distant metastasis. (a) Heat map shows qRT-PCR results of the downregulated downstream target genes of either the β-catenin or TGF-β signaling in miR-128-3p-inhibited (Anti-miR) cells, compared with control cells (NC). (b) Mice bearing tumor xenografts by subcutaneous inoculation (i.s.) or experimental metastasis by intravenous injection (i.v.) of A549-luc-CDDP-4th cells transduced with constitutively active β-catenin and SMAD3 mutants, alone or in combination, were intravenously administered with miR-128-3p antagomir, and mice bearing tumor xenografts (left panel) or experimental metastasis (right panel) of A549-luc-CDDP-4th cells were intraperitoneally injected with ICG-001 and LY2157299, alone

or in combination. Bioluminescent images of subcutaneous tumors and spontaneous/experimental metastasis are shown. (c) Immunostaining of β-catenin, SMAD3, E-cadherin, Vimentin, CD34 and TUNEL-positive apoptotic cells in tumor tissues of the A549-luc-miR-128-3p cell xenografts with the indicated treatments. For (c), representative images of CD34 and TUNEL-positive apoptotic cells were photographed by original x400 magnification; representative images of β-catenin, SMAD3, E-cadherin and Vimentin were photographed by original x400 magnification, which was further zoomed in.

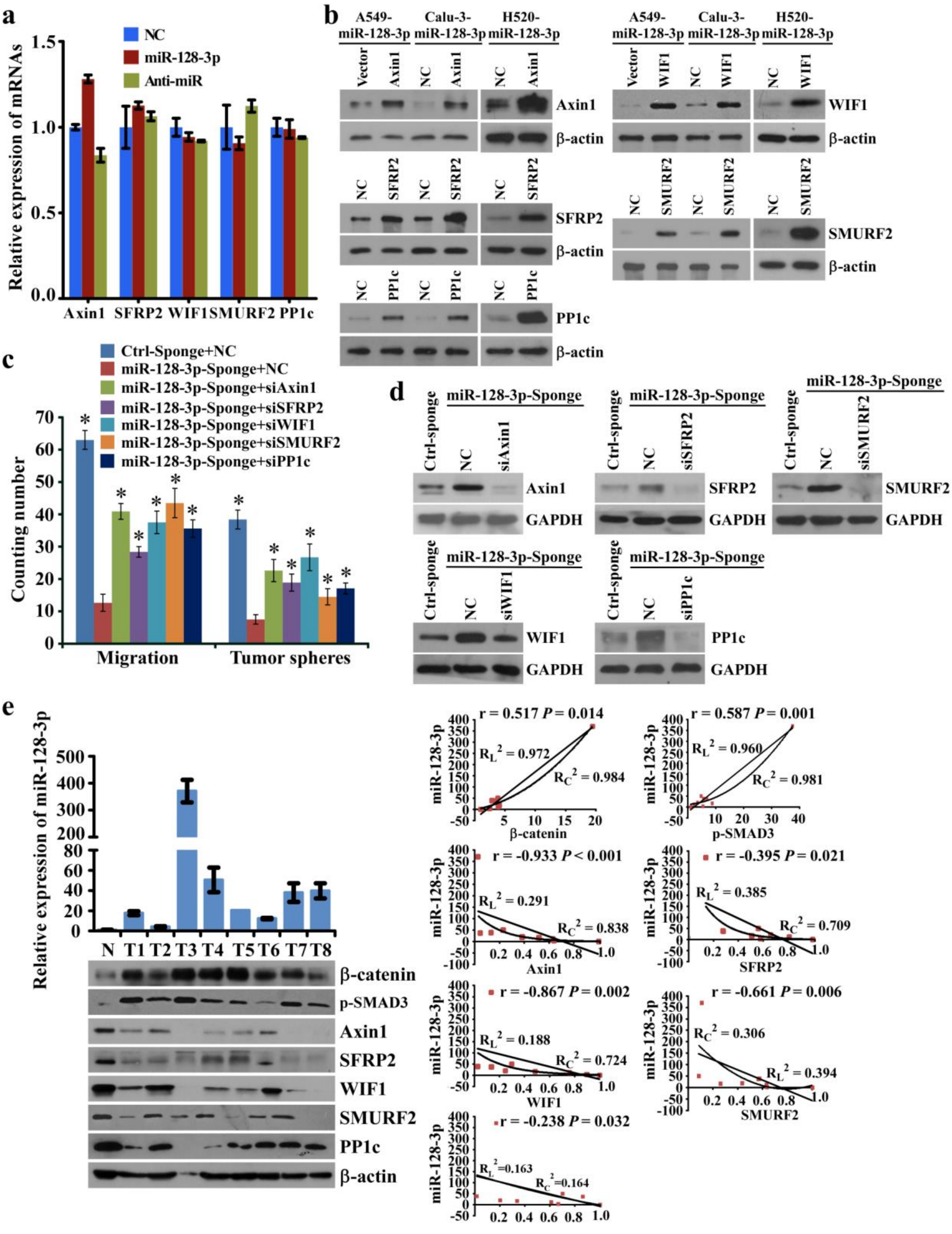

Supplementary Figure 9 miR-128-3p potently targets Axin1, SFRP2, WIF1, SMURFS or PP1c to activate the Wnt/β-catenin and TGF-β signaling in NSCLC cells. (a) qRT-PCR analysis of mRNA levels of Axin1, SFRP2, WIF1, SMURFS and PP1c in indicated cells. (b) WB analysis of the protein levels of Axin1, SFRP2, WIF1, SMURF2 or PP1c in miR-128-3p-overexpressing

cells with the indicated transfections. A two-tailed Student’s *t*-test was used for statistical analysis (* $P<0.05$). (c) Effects of silencing Axin1, SFRP2, WIF1, SMURFS or PP1c in A549-luc-miR-128-sponge cells on cell migration and growth of tumor cell spheres. (d) WB analysis of knockdown efficiency of indicated siRNAs in A549-luc-miR-128-sponge cells. (e) Expression and correlation analyses of miR-128-3p with β-catenin (non-phospho-(Ser33/37/Thr41)-β-catenin), phospho-SMAD3, Axin1, SFRP2, WIF1, SMURF2 and PP1c in 8 freshly collected NSCLC samples (T1-T8) normalized to non-cancerous lung tissue (N).

Supplementary Figure 10

Full unedited gel for Figure 2b and 2i

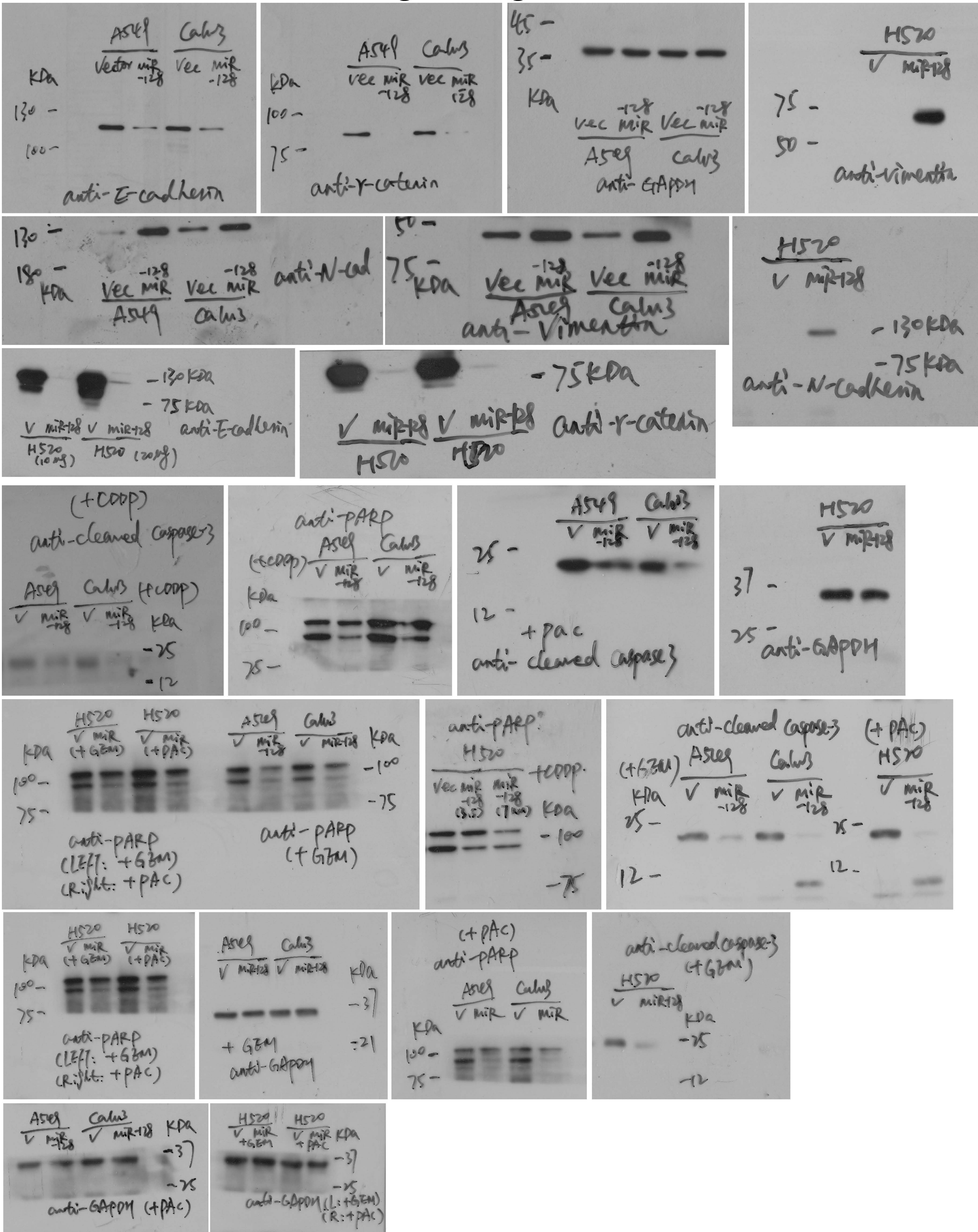

Supplementary Figure 10

Full unedited gel for Figure 7b

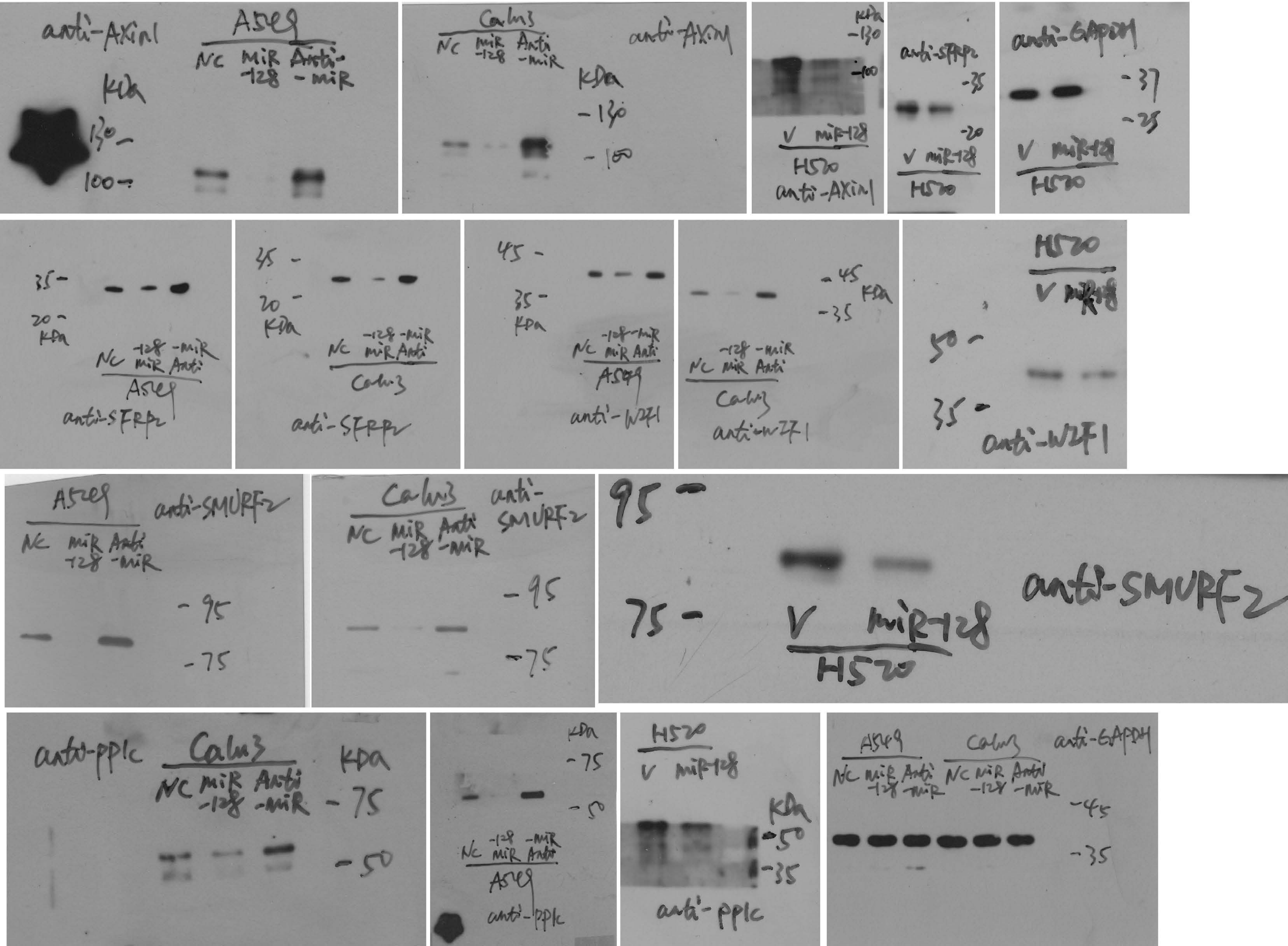

Supplementary Figure 10

Full unedited gel for Supplementary Figure 3g and 3l

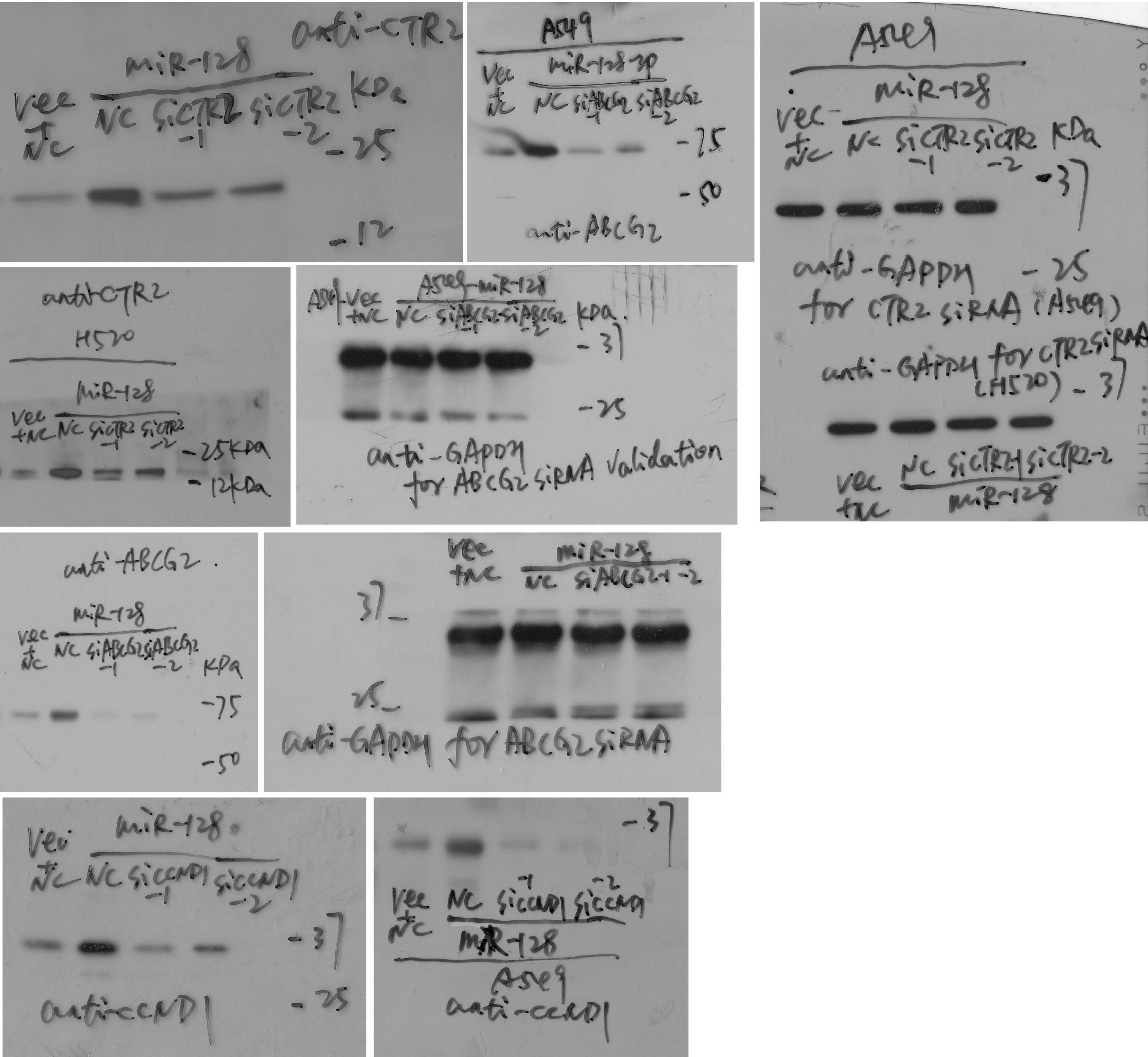

Supplementary Figure 10

Full unedited gel for Supplementary Figure 4b, 4k and 4l

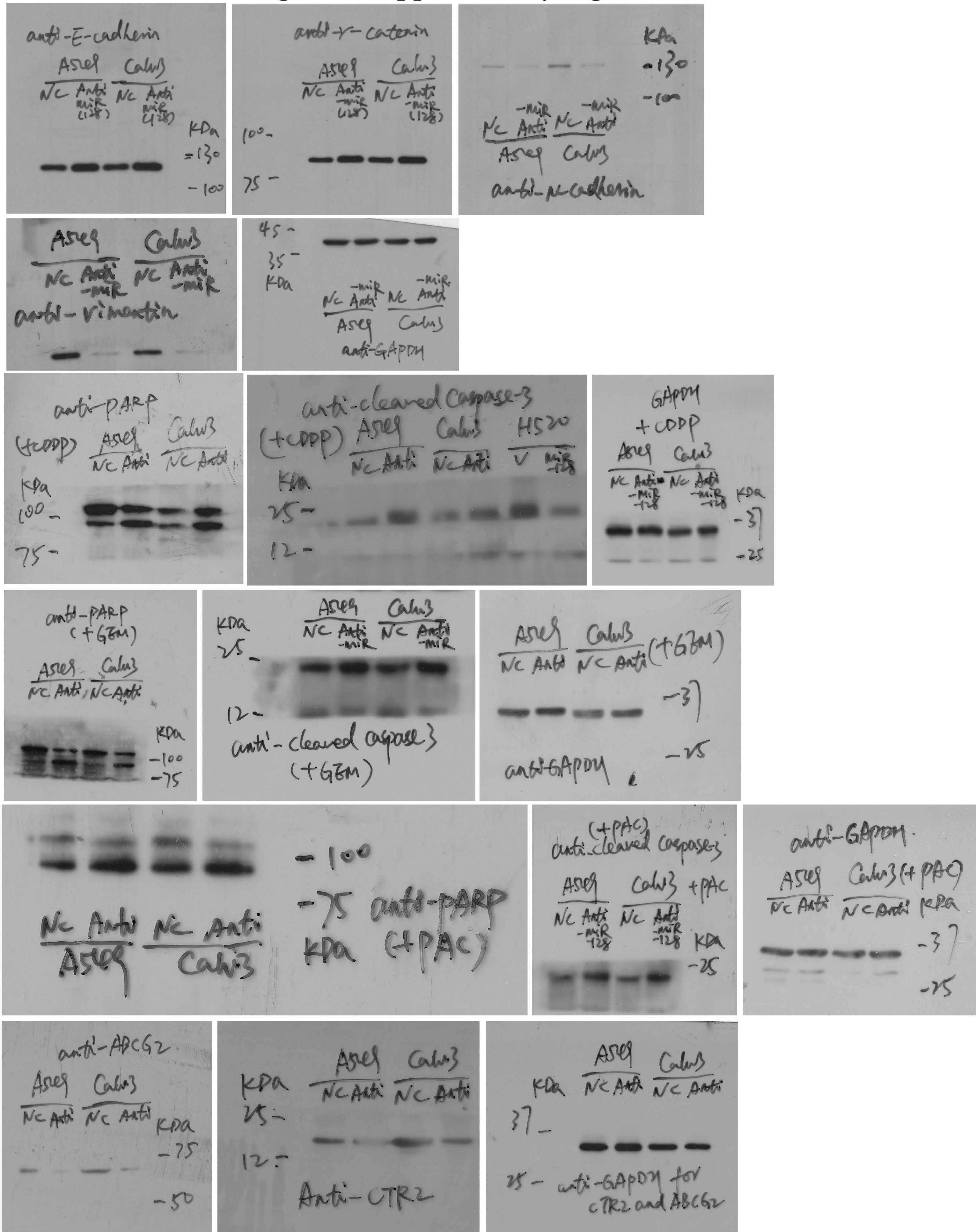

Supplementary Figure 10

Full unedited gel for Supplementary Figure 9b and 9e

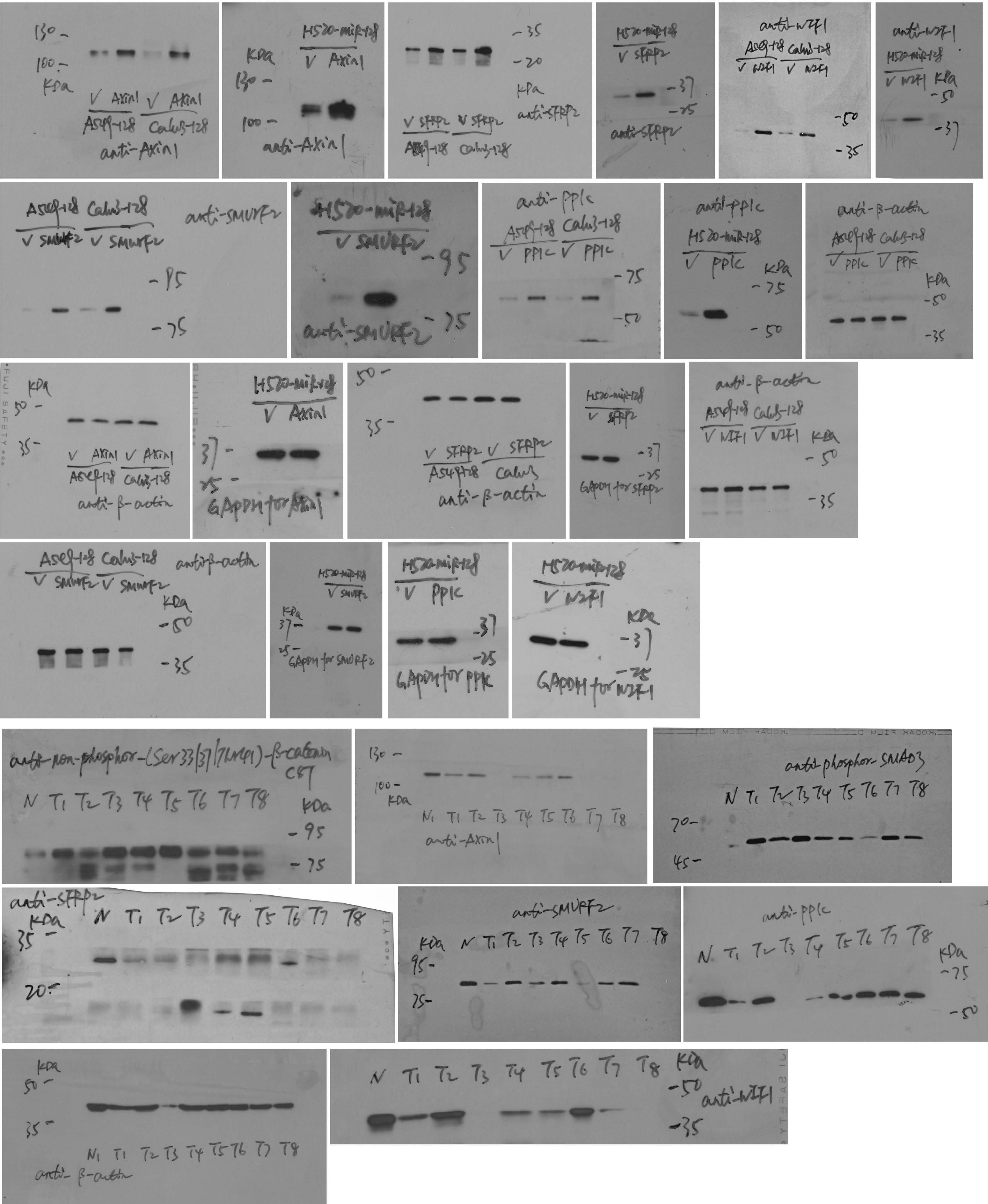

Supplement: Supplementary Information [file ncomms15870-s1.pdf]
